# Two‐Dimensional MoS_2_‐Based Anisotropic Synaptic Transistor for Neuromorphic Computing by Localized Electron Beam Irradiation

**DOI:** 10.1002/advs.202408210

**Published:** 2024-10-16

**Authors:** Lei Liu, Peng Gao, Mengru Zhang, Jiadu Dou, Chunsen Liu, Tuo Shi, Hao Huang, Chunlan Wang, Han He, Zijun Chen, Yang Chai, Jianlu Wang, Xuming Zou, Lei Liao, Jingli Wang, Peng Zhou

**Affiliations:** ^1^ State Key Laboratory of Integrated Chip and System Frontier Institute of Chip and System Fudan University Shanghai 200433 China; ^2^ School of Microelectronics Fudan University Shanghai 200433 China; ^3^ Zhejiang Laboratory Hangzhou 311122 China; ^4^ State Key Laboratory of Featured Metal Materials and Life‐cycle Safety for Composite Structures School of Resources Environment and Materials Guangxi University Nanning 530004 China; ^5^ School of Science Xi'an Polytechnic University Xi'an 710048 China; ^6^ Department of Applied Physics The Hong Kong Polytechnic University Hong Kong 999077 China; ^7^ College of Semiconductors (College of Integrated Circuits) Hunan University Changsha 410082 China

**Keywords:** anisotropic synapse, colored‐digit recognition, connection heterogeneity, electron beam irradiation, localized doping, multiterminal transistor, neuromorphic computing

## Abstract

Neuromorphic computing, a promising solution to the von Neumann bottleneck, is paving the way for the development of next‐generation computing and sensing systems. Axon‐multisynapse systems enable the execution of sophisticated tasks, making them not only desirable but essential for future applications in this field. Anisotropic materials, which have different properties in different directions, are being used to create artificial synapses that can mimic the functions of biological axon‐multisynapse systems. However, the restricted variety and unadjustable conductive ratio limit their applications. Here, it is shown that anisotropic artificial synapses can be achieved on isotropic materials with externally localized doping via electron beam irradiation (EBI) and purposefully induced trap sites. By employing the synapses along different directions, artificial neural networks (ANNs) are constructed to accomplish variable neuromorphic tasks with optimized performance. The localized doping method expands the axon‐multisynapse device family, illustrating that this approach has tremendous potentials in next‐generation computing and sensing systems.

## Graphical abstract

Neuromorphic computing provides a promising solution to the von Neumann bottleneck, paving the way for next‐generation computing systems. This study demonstrates an approach to realize anisotropic artificial synapses on isotropic materials via localized electron beam irradiation. Axon‐multisynapse systems are constructed to enhance the performance of artificial neural networks (ANNs). This method exhibits significant potentials for advanced computing and sensing system.

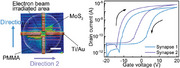

## 1 Introduction

In the era of big data, conventional computing systems, constrained by von Neumann architecture, face challenges in energy efficiency due to the segregation of computing and storage units.^[^ ^1^, ^2^, ^3^ ^]^ Inspired by the brain, neuromorphic computing offers a promising and energy‐efficient approach for developing advanced intelligent systems.^[^ ^4^, ^5^, ^6^ ^]^ The axon‐multisynapse architecture, where each axon connects to multiple synapses, plays a crucial role in neural information processing and complicated brain function within the neural network.^[^ ^7^, ^8^ ^]^ This architecture enables neurons to transmit information simultaneously to multiple target neurons or regions, facilitating the integration and coordination of diverse neural signals for more efficient processing. Connection heterogeneity induced by the intrinsic heterogeneity in axon‐multisynapse system allows diverse responses to the same signal, increasing the structural complexity and functional diversity of neural network.^[^ ^9^, ^10^, ^11^, ^12^ ^]^ This diversity enables sophisticated tasks to be accomplished through cooperation with other properties in brain such as perception, motor control, and cognitive processes. Furthermore, connection heterogeneity optimizes the performance of synaptic plasticity, improving the adaptability and learning capacity of neural networks in responding to dynamic external environments.^[^ ^13^, ^14^, ^15^ ^]^ Thus, realizing artificial synapses with connection heterogeneity is a crucial step for achieving neuromorphic computing with high complexity and realizing sophisticated brain functions.

Recent developments in axon‐multisynapse system based on anisotropic 2D materials exhibit its great potential to mimic the human brain behaviors. Tian et al. first achieved the axon‐multisynapse network on anisotropy 2D black phosphorus with compact device structure (BP).^[^ ^9^ ^]^ Qin et al. further illustrated that synaptic transistor based on anisotropic selenium (t‐Se) can act as a filter with low power consumption.^[^ ^10^ ^]^ These works provide feasible approaches to construct axon‐multisynapse system with desired connection heterogeneity. However, in the context of prior research, the construction of the axon‐multisynapse system has predominantly depended on the inherent anisotropy of the channel material. The inflexible anisotropy ratio and band structure have imposed constraints on its applicability. Furthermore, a significant proportion of anisotropic 2D materials either lack scalability in their preparation or exhibit instability in atmospheric conditions.^[^ ^16^, ^17^ ^]^ These challenges have curtailed the potential applications of artificial anisotropic synapses.

In this work, we employ a localized electron beam irradiation (EBI) technique to induce anisotropic properties in an isotropic MoS_2_ synaptic device. The precisely controlled n‐type doping, a consequence of localized EBI, results in the MoS_2_‐based transistor demonstrating a variety of photoelectric characteristics in response to identical stimuli along different directions. With this approach, we can precisely tune the anisotropic synaptic behavior by modulating the EBI intensity. We further investigate connection heterogeneity in the MoS_2_‐based multiterminal synaptic transistor to realize an axon‐multisynapse system. By harnessing the synaptic plasticity in our artificial anisotropic synapse, we successfully execute tasks of image recognition and colored‐digit identification through the use of artificial neural networks (ANNs). The axon‐multisynapse system with varying orientations is fine‐tuned to maximize both the recognition speed and rate for their respective tasks. Our results offer a universal approach for the design and fabrication of axon‐multisynapse system.

## 2 Results and Discussion

### 2.1 Memory Features and Synaptic Functions of UVO‐Treated MoS_2_ Transistor

**Figure 1:**
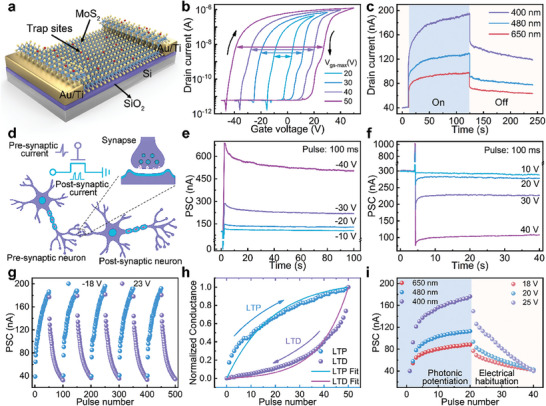
The memory properties and synaptic behaviors of MoS_2_‐based transistor after UVO treatment. a) Structure schematic of the MoS_2_‐based transistor. b) Transfer characteristics of the MoS_2_‐based transistor with the different *V_gs‐max_* (the maximum value of *V_gs_* sweeping range) at *V_ds_* of 0.5 V. c) The different responses of drain current under illumination with different wavelengths. d) Schematic illustration of the biological synapse and the MoS_2_‐based artificial synaptic device. The *V_ds_* is set as 0.1 V. e) EPSC generated under negative pulses with different amplitudes of −10, −20, −30, and −40 V. f) IPSC generated under positive pulses with different amplitudes of 10, 20, 30, and 40 V, respectively. g) Reproducible potentiation and depression processes under continuous negative pulses and positive pulses. h) The fitted nonlinearity coefficient of potentiation and depression processes. i) The change of PSC under optical and electric signals. The drain electrode is applied reading pulses of 0.1 V with a time interval of 3 s.

We first investigate the electrical and optical memory behavior of the MoS_2_‐based synaptic device with axon‐singlesynapse structure, which will be turned into axon‐multisynapse later in the manuscript. **Figure** **1**a presents a schematic structure of a back‐gate transistor constructed from MoS_2_. The introduction of trap sites on the MoS_2_ surface is facilitated through the use of ultraviolet/ozone (UVO). Figure S1 (Supporting Information) illustrates the Raman characteristics of MoS_2_ films before and after the UVO treatment. Figure 1b presents the double sweep transfer curves of the MoS_2_‐based transistor with various swept ranges of gate voltage (*V_gs_*) after UVO treatment. The threshold voltage (*V_th_*) for the forward sweep is lower as compared to reverse sweep, resulting in clockwise hysteresis. The hysteresis window can be tuned from 13 to 64 V with the increase of maximum *V_gs_* from 20 to 50 V. This suggests that the charge trapping behavior can be modulated by adjusting the maximum value of the *V_gs_*. Figure S2 (Supporting Information) explores double sweep transfer characteristics of the device with different irradiation time of UVO treatment. Figure S3 (Supporting Information) presents the transfer characteristics of UVO‐treated MoS_2_ transistor under various drain voltage (*V_ds_*) and corresponding output characteristics under varying *V_gs_*. Statistic characteristics of 30 devices are given in Figure S4 (Supporting Information). Continuous changes of drain current under different gate pulse and retention properties are given in Figure S5 (Supporting Information). Subsequently, we investigate the optical modulation of the UVO‐treated MoS_2_ transistor under illumination with wavelengths of 650, 480, and 400 nm (30 mW cm^−2^). Figure 1c shows the photoresponse of MoS_2_ transistor during a 110 s illumination period, followed by a 120 s post‐illumination recovery phase. Under continuous illumination, the drain current exhibits a gradual increase over time, indicative of a current excitation characteristic. The drain current increases from 40 nA to ≈ 97, 129, and 195 nA under the *V_ds_* of 0.1 V, corresponding to light wavelengths of 650 nm, 480 nm and 400 nm, respectively. Then the light‐induced current will drop steeply and stabilize at ≈ 63 (650 nm), 77 (480 nm), and 118 nA (400 nm) after light illumination, indicating the nonvolatility responding to optical signals. The memory characteristics of the MoS_2_‐based transistor is attributed to the trap sites generated after UVO treatment.^[^ ^18^, ^19^ ^]^ Figure S6 (Supporting Information) elucidates the band diagram and the underlying mechanism of this memory behavior.

The distinct memory behaviors exhibited by the UVO‐treated MoS_2_ transistor inspire further investigation into the artificial synaptic characteristics of this device. The artificial synapse mimics biological synapses^[^ ^20^, ^21^, ^22^ ^]^ by generating a corresponding postsynaptic current (PSC) in response to pulse signals applied at the gate as presynaptic stimulation (Figure 1d). Figure 1e,f illustrate the synaptic response to gate pulses of varying amplitudes. The MoS_2_‐based artificial synapses simulate excitatory PSC (EPSC) under negative presynaptic signals and inhibitory PSC (IPSC) under positive ones. Figure S7a,b (Supporting Information) show EPSC behaviors in response to pulses with different widths and frequencies. Continuous electrical pulse trains are used to modulate synaptic weight in Figure 1g. The PSC is progressively enhanced or suppressed under negative or positive pulse trains (Figure S7c, Supporting Information), mirroring biological long‐term potentiation (LTP) and long‐term depression (LTD), respectively. The PSC is adjusted over five cycles of negative and positive pulse sequences, demonstrating the high repeatability of these artificial synapses. The nonlinearity factor (NLF)^[^ ^23^, ^24^ ^]^ is fitted according to the behavioral model (See Note S1, Supporting Information), as indicated in Figure 1h. The MoS_2_‐based artificial synapse presents the NLF of 3.19 for LTP and 4.34 for LTD. The retention property after pulse sequence is investigated in Figure S7d (Supporting Information). Based on the long‐term plasticity of MoS_2_ artificial synapses, the ANN model is constructed to conduct pattern recognition, as shown in Figures S8 and S9 (Supporting Information). Figure 1i presents the wavelength dependent optical potentiation and electrical habitation characteristics of the MoS_2_ synaptic device. During the illumination, drain current increases more quickly with a shorter wavelength. The light‐induced PSC are 337.64%, 183.79%, and 119.96% for 400, 480, and 650 nm, respectively. The light with shorter wavelength generates more additional electrons into the channel, larger electrical pulse is needed to reset the PSC to initial state. In order to balance the potentiation and habitation behavior of the optical and electrical pulse, the electrical pulse amplitude is set to be 25 (400 nm), 20 (480 nm), and 18 V (650 nm). These results illustrate the potential of the UVO‐treated MoS_2_ transistor in neuromorphic computing systems, paving the way for the development of axon‐multisynapse system later in this study.

### 2.2 Localized EBI Method to Realize Anisotropic Conductivity

**Figure 2:**
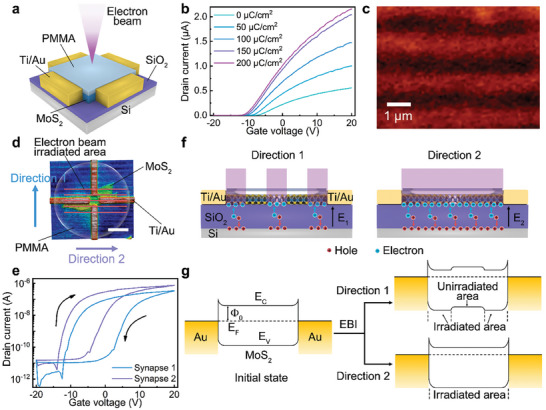
The anisotropic behaviors and mechanisms of MoS_2_‐based transistor treated by localized EBI. a) Schematic of the device treatment procedures by electron beam. b) Transfer characteristics of primordial MoS_2_‐based transistor after EBI treatment on the whole channel with different area doses at *V_ds_* of 0.5 V. c) Mapping image of PL peak center of the MoS_2_ nanoflake after localized EBI. d) Schematic of the anisotropic multiterminal synaptic device irradiated by localized electron beam. Synapse 1 and synapse 2 are represented by devices along direction 1 and direction 2, respectively. The scale bar is 10 µm. The channel length and width are 6 and 3 µm, respectively. e) Transfer characteristics of the synapses 1 and 2 at the *V_ds_* of 0.5 V. f) Schematic diagrams to illustrate the n‐type doping induced by electron beam irradiation. g) Energy band diagrams of the MoS_2_ transistor before and after EBI.

We subsequently use localized EBI to achieve anisotropic conductivity of MoS_2_ for axon‐multisynapse system. **Figure** **2**a illustrates the experimental scheme while Figure S10 (Supporting Information) details device fabrication procedures. A polymethyl methacrylate (PMMA) film is spin‐coated onto the device as a protective layer to prevent the deposition of impurities and electron‐beam‐generated reactions on the MoS_2_ surface. Figure S11 (Supporting Information) presents the comparison of transfer curves of the devices before and after the spin‐coating process. Doping is accomplished by EBI treatment in e‐beam lithography system with an energy of 10 keV.^[^ ^25^ ^]^ We first investigate the carrier concentration tuning ability of the EBI doping method. Figure 2b shows the transfer characteristics of MoS_2_ transistor after EBI across the entire device. Initially, the MoS_2_‐based transistor is undoped with a *V_th_* of ≈−6.93 V. *V_th_* gradually shifts negatively with increasing e‐beam dose. Eventually, *V_th_* shifts to −10.49 V and the overall current increases over 4 times after EBI with an area dose of 150 µC cm^−2^. Further increasing the e‐beam dose to 200 µC cm^−2^ shows little influence to the carrier and current density. The carrier concentration *n* is calculated as:^[^ ^26^ ^]^

(1) $$n=\frac{C_{0}\left|V_{gs}-V_{th}\right|}{\left|q\right|}\;$$

where *C_0_* is the gate capacitance per unit area and is 34.5 nF cm^−2^ for 100 nm SiO_2_, |*q*| = 1.6 × 10^−19^ C is the elementary charge and *V_th_* is the threshold voltage. The carrier concentration of MoS_2_ at the *V_gs_* of 0 V is tuned from 1.49 × 10^12^ cm^−2^ to 2.37 × 10^12^ cm^−2^ with 200 µC cm^−2^ EBI treatment. After being treated by EBI, the electron‐hole pairs are generated in the SiO_2_ layer. Subsequently, electrons will be trapped at the interface between MoS_2_ and SiO_2_. Holes will accumulate at the interface between Si and SiO_2_. As a result, an electrostatic field is building up to form the n‐type doping effect on the MoS_2_. The above results indicate that carrier concentration can be well controlled by the EBI treatment. Utilizing the precisely control of the EBI with e‐beam lithography, the EBI doping method not only allows for precisely control of the carrier concentration but also the doping area. We use photoluminescence (PL) and Raman mapping to image the pattern we predefined. Figure 2c shows PL mapping image of a stripe pattern drawn using the e‐beam lithography. The PL image clearly reveals a striped modulation resulting from the different localized carrier density.^[^ ^27^, ^28^ ^]^ Due to the resolution limitation of the PL mapping, the width of the stripe is limited to 1 µm. We remark that the feature size of this doping method can be even smaller due to the high resolution of the e‐beam lithography. The uniform FWHM of Raman mapping before and after EBI indicates that this doping method doesn't introduce defects or disorder into MoS_2_ lattice (Figure S12, Supporting Information).

Benefiting from the localized precisely modulation of MoS_2_ carrier density, we achieve anisotropic characteristics by fabricating periodic highly conductive stripe^[^ ^29^ ^]^ on isotropic MoS_2_ device with EBI. As depicted in Figure 2d, a UVO‐treated MoS_2_ transistor is subjected to a periodically localized EBI. The irradiated direction is denoted by the red line (1 µm line width). Figure 2e displays the double sweep transfer characteristics with an on/off ratio of ≈ 10^6^ along direction 1 (synapse 1) and along direction 2 (synapse 2) after the localized EBI treatment with an area dose of 150 µC cm^−2^. The UVO‐induced memory properties are preserved after EBI. Compared to synapse 1, the drain current of synapse 2 is 2 times higher than synapse 1, illustrating that anisotropic features are achieved by the periodically localized EBI. The mechanism of the anisotropic characteristics is proposed in Figure 2f. The regions exposed and unexposed to the electron beam exhibit distinct doping concentrations, which is caused by the localized electron‐hole pairs generated in the SiO_2_ layer.^[^ ^30^, ^31^ ^]^ To further illustrate the anisotropic properties, the energy band diagrams are proposed in Figure 2g. After EBI treatment, n‐type doping will enable the Fermi level (E_F_) shift closer to the conduction band in the irradiated area. In synapse 1, these two regions constitute periodically n‐n^+^ junction, while in synapse 2, they are arranged in parallel. The highly conductive stripes increase the conductivity of synapse 2. However, the n‐n^+^ junction and the less conductive unexposed area limit the conductivity of synapse 1.

### 2.3 Adjustable Anisotropy Ratio Achieved by Modulating EBI Intensity

**Figure 3:**
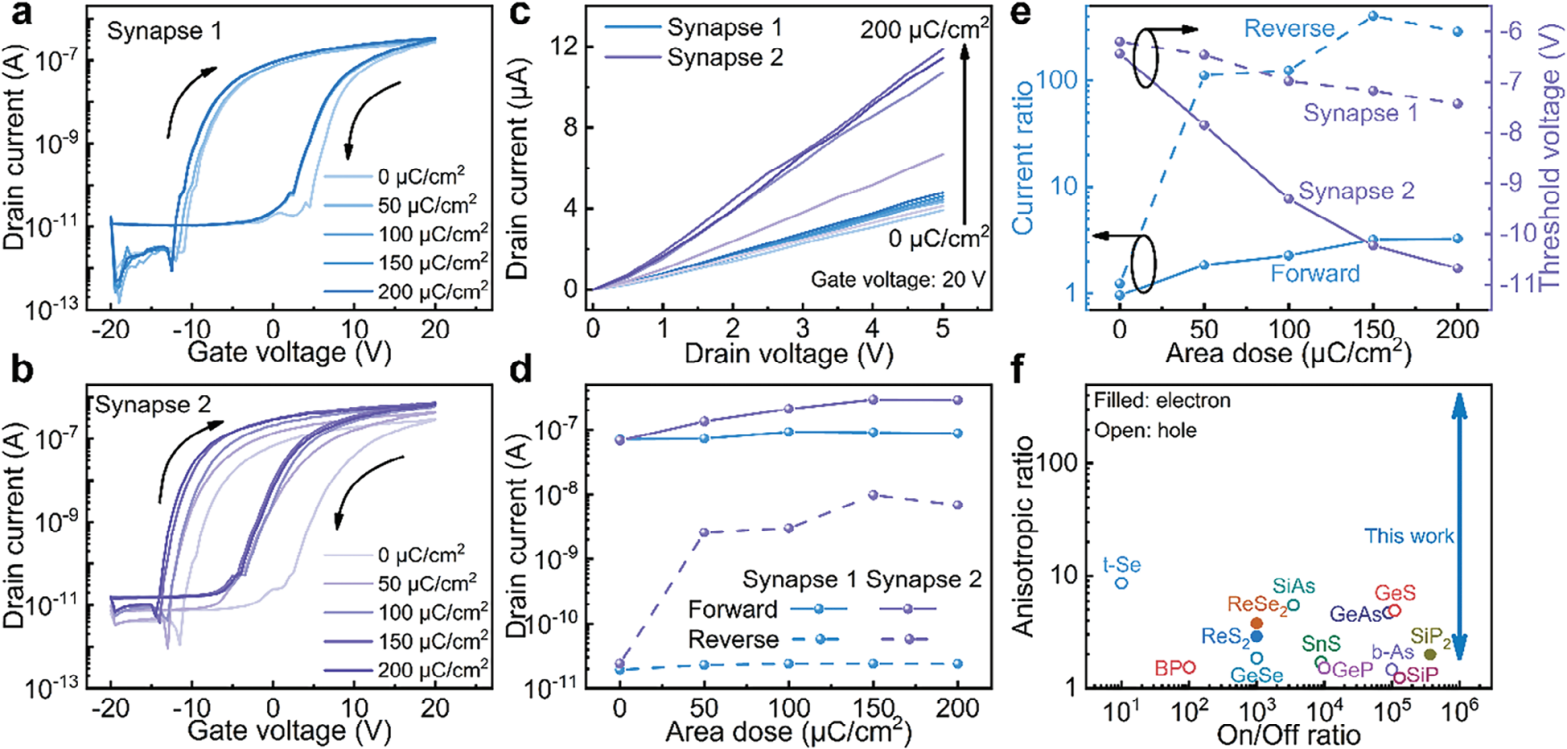
The anisotropic properties of MoS_2_‐based multiterminal transistor after EBI treatment with different area doses. Properties of the transfer characteristics of MoS_2_‐based transistor after EBI treatment with different area doses at *V_ds_* of 0.5 V for a) synapse 1 and b) synapse 2. c) Output characteristics after EBI treatment with different area doses at *V_gs_* of 20 V for synapse 1 and synapse 2. d) Drain current extracted from transfer curves of both synapses under zero gate bias. e) The current ratio and threshold voltage of both synapses with different EBI doses. f) Comparison of anisotropic ratio and on/off ratio of 2D anisotropic device.

We further investigate the anisotropic electrical properties of MoS_2_‐based multiterminal transistor with various EBI intensities. **Figure** **3**a,b shows the double sweep transfer characteristics along two directions with the increasing area doses from 0 to 200 µC cm^−2^. For n‐channel MoS_2_ device, electron trapped at absorptive sites is suppressed at large negative *V_gs_*, which refers to the forward sweep starting from negative side. For the forward sweep in Figure 3a, the *V_th_* shows a little negative shift of 1.23 V from ‐6.20 V to ‐7.43 V after 200 µC cm^−2^ EBI for synapse 1. During forward sweep, the traps are not occupied, and the conductivity is closer to the characteristics without charge trapping behavior. As discussed previously, the conductivity of synapse 1 is mainly determined by the unexposed area, although the EBI continuously increases the conductivity of the highly doped stripe, the high conductive region shows little influence to the conductivity of this direction. On the contrary, for the forward sweep in Figure 3b, the *V_th_* shows negative shift of 4.24 V from −6.44 to −10.68V for synapse 2 with the same EBI intensity which is much larger than synapse 1. As discussed previously, the conductivity of synaptic 2 is mainly determined by the exposed area. With the EBI intensity increasing, the conductivity of exposed area increases, along with the conductivity of synapse 2. Furthermore, the hysteresis window is 14.5 V for synapse 1 before EBI treatment and decreases to 13.8 V after EBI with an area dose of 200 µC cm^−2^. In contrast, the hysteresis window of synapse 2 is reduced from 13 to 10 V under the same condition, demonstrating a more significant change compared to synapse 1. This anisotropic behavior is attributed to the nonuniform distribution of trap sites in the MoS_2_ channel, which affects the memory characteristics of the MoS_2_‐based transistor. EBI treatment induces n‐type doping, which results that trap sites are occupied by generated electrons. The variation in electron distribution between direction 1 and direction 2 results in different numbers of occupied traps. Figure 3c is the *I*–*V* characteristics of both synapses with different EBI intensities under 20 V *V_gs_*. The device shows a slight increase by only 0.87 µA for synapse 1. The increase is caused by the reduction of resistance in exposed area, and the small value is attributed to the large resistance of the unexposed area. The current increases from 4.13 to 11.43 µA by ≈2.77 times for synapse 2, which matches the transfer curve well. The drain current for both synapses under 0 V *V_gs_* of forward curves and reverse curves are plotted in Figure 3d, exhibiting the different tendencies with increasing dose. The current of synapse 1 shows little changes under all doses and the current of synapse 2 shows the highest value under 150 µC cm^−2^. In particular, the reverse current of synapse 2 is enhancing from 2.42^−11^ A to 9.79^−9^ A at 0 V *V_gs_*. The current isn't increasing while the dose is further raised. The current ratio of these two directions and *V_th_* with different e‐beam doses are given in Figure 3e. The current ratio increases with the e‐beam dose. The current ratios range from 0.96 to 3.28 for forward curves and range from 1.24 to 406.40 for reverse curves. Figure 3f demonstrates the comparison of anisotropic MoS_2_‐based transistor with other 2D anisotropic device,^[^ ^9^, ^10^, ^11^, ^32^, ^33^, ^34^, ^35^, ^36^, ^37^, ^38^, ^39^, ^40^, ^41^, ^42^ ^]^ exhibiting high on/off ratio and adjustable anisotropic ratio.

### 2.4 Simulation of Connection Heterogeneity in Axon‐Multisynapse System

**Figure 4:**
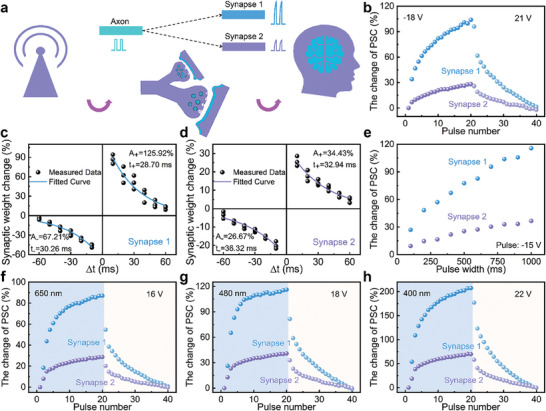
The anisotropic synaptic behaviors of the MoS_2_‐based multiterminal transistor. a) Biological axon‐multisynapse system with connection heterogeneity. b) Conductance modulation of the device for synapse 1 and synapse 2. Anisotropic STDP behaviors for c) synapse 1 and d) synapse 2. e) The change of PSC under different pulse widths for synapse 1 and synapse 2. The anisotropic change of PSC under optical and electric signals of f) 650 nm and 16 V, g) 480 nm and 18 V, h) 400 nm and 22 V.

According to the anisotropic electrical properties of the MoS_2_‐based multiterminal transistor after EBI, axon−multisynapse system is constructed to simulate the connection heterogeneity, which is significant feature to achieve diversity of neurological events. As shown in **Figure** **4**a, external environmental signals are perceived and then transmitted through the axon to the synapse. The identical signal from the axon generates various responses at postsynapses because of connection heterogeneity. These responses are eventually processed to accomplish complicated functions like perception, learning, memory, oblivion, and so forth along with other properties in the brain. Before measurement, both synapse 1 and synapse 2 are adjusted to the respective initial state by applying the same sequence of 50 pulses with the amplitude 20 V. Figure S13 (Supporting Information) presents the anisotropic EPSC and IPSC responses of synapse 1 and synapse 2. Figure 4b demonstrates the various changes of PSC (ΔPSC/PSC) along directions 1 and 2 under negative and positive pulse trains. After the same 20 negative pulses, the change of PSC will gradually increase to 103.98% in synapse 1 and 28.11% in synapse 2. Then PSC of both synapse 1 and synapse 2 is restored to the initial state under 20 positive pulses. Figure S14 (Supporting Information) shows corresponding fitted nonlinearity coefficient of synapse 1 and synapse 2. These results indicate that the change of synaptic weight in synapse 1 is significantly larger than that in synapse 2. The anisotropic synaptic plasticity results from the difference in drain current along the two directions induced by EBI. Before applying stimulus signal at gate, the initial current of synapse 2 is larger comparing to that of synapse 1. Due to the identical voltage spike from gate, the number of trapped charges is approximately equal, resulting the similar ΔPSC but different change of PSC in the two artificial synapses.

Spiking timing dependent plasticity (STDP) is an essential learning characteristic of long‐term plasticity, in which the synaptic weight can be modulated by the time intervals (*Δt*) between pre‐ and post‐synaptic spikes.^[^ ^43^ ^]^ As shown in Figure S15 (Supporting Information), spike pairs are designed to realize STDP characteristics. Figure 4c,d demonstrates the change of synaptic weight responding to the varied *Δt* in synapse 1 and synapse 2, which is consistent with the asymmetric Hebbian STDP rule in biology. According to the relationship between *W_Change_* and *Δt* in asymmetric Hebbian STDP (See Note S2, Supporting Information), A_+_, A_‐_, τ_+_, and τ_‐_ are ≈125.92%, ≈67.21%, ≈28.70 ms, and ≈30.26 ms for synapse 1 and ≈34.43%, ≈26.67%, ≈32.94 ms and ≈36.32 ms for synapse 2, which indates the dissimilarity of STDP behaviors in these two synapses. Figure 4e depicts the anisotropic PSC change reponding to variable pulse widths with fixed amplitude of ‐15 V. Equally, the change of drain current along direction 1 is larger than that along direction 2. The optical responses for the two synapses under illumination with various optical wavelengths are presented in Figure S16 (Supporting Information). Subsequently, photonic potentiation and electric habituation are further investigated for the anisotropic synapses, as shown in Figure 4f–h. Also, reading pulses of 0.1 V are applied to the drain electrode with a time interval of 3 s when illuminated. The PSC will increase gradually responding to optical stimulation with different wavelengths. And the change of PSC in synapse 1 is also larger comparing to synapse 2. After light illumination, positive voltage pulses with the amplitude of 16 V, 18 V, and 22 V are applied to restore the light‐induced PSC, responding to the light wavelengths of 650, 480, and 400 nm, respectively. The anisotropic response to optical stimulation also results from the differing initial currents in the two directions. When exposed to light, photoinduced carriers are generated in the MoS_2_‐based channel. Synapses 1 and 2 exhibit comparable ΔPSC as a result of the MoS_2_ layer's essentially constant quantity of photoinduced carriers in response to light illumination in both directions. Whereas due to the difference of initial currents in these two directions, the multiterminal synaptic transistor exhibits anisotropic change of PSC in response to light illumination. These findings provide the feasible approach constructing axon−multisynapse system to accomplish various complicated functions in brain.^[^ ^44^, ^45^, ^46^ ^]^

### 2.5 Image Recognition and Colored‐Digit Recognition Based on Anisotropic Synaptic Features

**Figure 5:**
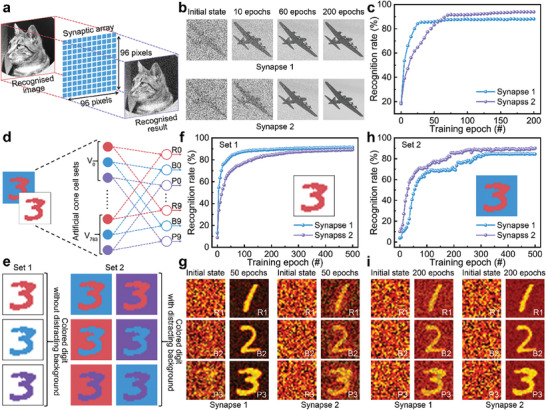
Neuromorphic computing by the neural networks based on the axon−multisynapse system. a) The schematic of ANN to accomplish image recognition. b) Mapping synaptic weights of the input image for synapse 1 and synapse 2 after different learning epochs. c) The comparison of image recognition accuracy of synapse 1 and synapse 2. d) The schematic of ONN to recognize colored digits. e) MNIST handwritten datasets for colored‐digit recognition. Set 1 consists of colored digits without distracting background, and Set 2 is composed of colored digits with distracting background. f) The recognition accuracy of the synapse 1 and synapse 2 to recognize colored digits without distracting background. g) Mapping synaptic weights of colored digit for synapse 1 and synapse 2 at initial state and after 50 epochs trained by Set 1. h) The recognition accuracy of the synapse 1 and synapse 2 to recognize colored digits with distracting background. i) Mapping synaptic weights of colored digit for synapse 1 and synapse 2 at initial state and after 200 epochs trained by Set 2.

Based on the anisotropic synaptic plasticity of axon−multisynapse system, an ANN is established to carry out image recognition, as illustrated in **Figure** **5**a. The 96 × 96 pixels recognized image is from STL‐10 dataset after greyscale processing.^[^ ^47^ ^]^ Subsequently, feature maps are extracted through an 18‐layer convolutional neural network (ResNet18)^[^ ^48^ ^]^ and then passed to the fully connected network for classification. Schematic diagram of this convolutional neural network is shown in Figure S17 (Supporting Information). In emulation process, weight updates are simulated by the LTP/LTD properties of synapse 1 and synapse 2, respectively. Figure 5b demonstrates the progression of the output image after different epochs during the learning process. It is observed that the ANN learned by the synaptic plasticity of synapse 1 displays more rapid learning effects, exhibiting the emergence of object outline after fewer learning epochs. Nevertheless, the ANN learned by the LTP/LTD features of synapse 2 shows improved accuracy, delivering clearer object details in the ultimate state. As presented in Figure 5c, the recognition accuracy of ANN based on synapse 1 is superior up to 56 epochs, but the recognition accuracy of ANN based on synapse 2 surpasses it after 56 epochs. These results suggest that the ANN based on synapse 1 enables faster image recognition, while the ANN based on synapse 2 achieves higher accuracy in the long run.

Moreover, a dual‐layer optoelectronic neural network (ONN) with colored identification function is constructed to effectively accomplish pattern recognition tasks according to the photoelectric synaptic behaviors of anisotropic MoS_2_‐based transistor, as presented in Figure 5d. The input layer is formed by 784 cone cell sets, and each set comprises 3 input neurons, exhibiting distinctive responses to red (R), blue (B), and purple (P) light. The colored lines are the artificial synapses with synaptic plasticity in various R/B/P weight regions. These sets of neurons generate varied synaptic dynamics in response to the input colored‐digit image and the 30 output neurons are used to export the results of R0‐R9, B0‐B9 and P0‐P9. MNIST handwritten dataset^[^ ^49^ ^]^ is employed for colored‐digit recognition after some modifications, as depicted in Figure 5e. The first set consists of colored digits without distracting background (Set 1) and the other is composed of colored digits with distracting background (Set 2). More details about the datasets’ origin, the number of images, the classification categories, and preprocessing steps are illustrated in Figures S18 and S19 (Supporting Information). During simulation, colored backgrounds are considered as visual distractions to interfere with the recognition of digits. Figure 5f shows recognition accuracy of the ONN for colored digits in Set 1. Following 50 learning epochs, the ONN associated with synapse 1 achieves the recognition accuracy of 83.11%, whereas the recognition accuracy of ONN linked with synapse 2 is 69.75%. In Figure 5g, the visualization of synaptic weights for both synapse 1 and synapse 2 is presented. The clearer delineation of digital patterns after trained by Set 1 illustrates the improved proficiency in recognizing colored digits. Furthermore, the visualization of synaptic weights of synapse 1 exhibits greater clarity compared to synapse 2, suggesting better performance of the ONN associated with synapse 1 for colored digit recognition without visual distractions originating from the background. Meanwhile, the recognition of colored digits in Set 2 is also carried out. Figure 5h demonstrates the ONN connected by synapse 1 and synapse 2 acquires the accuracy rates of 69.86% and 85.31% after 200 learning epochs, respectively. Post‐training visualization with synaptic weights of synapse 1 and synapse 2 by Set 2 confirms the enhance of recognition capability for colored digits, as shown in Figure 5i. Particularly, the mapping image of synapse 2 displays more distinguishable digit patterns against the background, indicating the superior performance for the colored‐digit recognition with visual distractions from the background. The above results indicate that synapses in both directions of axon−multisynapse system have enormous potential for neuromorphic computing responding to different demands.

## 3 Conclusion

In summary, we have successfully induced anisotropic properties in an isotropic material using a localized EBI method. The anisotropic transistor composed 2D MoS_2_ nanoflakes is fabricated after localized EBI treatment, exhibiting diverse responses when stimulated by the same either optical or electrical stimulus along different directions. Benefiting from modulation of area doses during EBI process, the multiterminal synaptic transistor with adjustable anisotropy is further realized. Axon−multisynapse system with connection heterogeneity in neural networks is simulated. Artificial synapses in axon−multisynapse system demonstrate variable synaptic plasticity along different directions. Eventually, based on the synaptic plasticity of multiterminal synaptic transistor, the ANN and ONN are constructed to execute neuromorphic tasks, including image recognition and colored‐digit recognition. Responding to various neuromorphic tasks, the synapses in variable directions are employed to enhance recognition performance. These findings provide feasible approaches to simply structure and accomplish complicated functions for neuromorphic computing system.

## 4 Experimental Section

### Fabrication of MoS_2_‐Based Transistor

The source and drain electrodes (Ti/Au, 7 nm/30 nm) are fabricated on the Si wafer with 100 nm SiO_2_ via photolithography (MA/BA Gen8, Suss Micro Tec, Germany), electron‐beam evaporation (BE400, De Technology Co., Ltd., USA) and lift‐off process. By preparing the device in this way, the MoS_2_‐based channel is more fully exposed so that it is treated more efficiently by UVO and EBI. The channel length and width are 6 µm and 3 µm, respectively. Then MoS_2_ nanoflakes (SixCarbon Technology, China) are mechanically exfoliated onto the top of source and drain electrodes to form the channel of transistor. Subsequent to the exfoliation process, device is annealed at 120 °C under vacuum conditions for 2 h by using vacuum heating stage.

### Process of UVO Treatment

The device is treated by UVO treatment (UV Ozone Cleaning System, CIF International Group Co., Ltd., China) at 100 °C for 90 s to generate trap states.

### Process of EBI Treatment

PMMA film is uniformly prepared onto the MoS_2_‐based transistor as protection layer via the spin‐coating process of 600 rpm for 10 s and 6000 rpm for 60 s. The PMMA‐coated device is then heated at 180 °C for 3 min to remove residual solvents. Finally, device is treated by EBI in e‐beam lithography system (Zeiss sigma 300 & Raith Elphy Quantum), wherein the area dose of the EBI treatment is set at 150 µC cm^−2^.

### Characterization of Devices

The electrical and photoelectric characteristics are measured by KEITHLEY 2636A sourcemeter. All transfer curves are measured at a scan speed of 1 V s^−1^. The light sources used in optical‐electrical measurements are 400, 480, and 650 nm, respectively (30 mW cm^−2^). These measurements are carried out in the vacuum environment at room temperature by using a vacuum probe stage (CGO‐4, Shenzhen Cindbest Instrument Equipment Co., Ltd., China).

## Conflict of Interest

The authors declare no conflict of interest.

## Author Contributions

L.L. and P.G. contributed equally to this work. J.W. and L.L. conceived and designed the project, analyzed the data, and wrote the manuscript. L.L. fabricated the devices and performed the measurement and the data analysis. G.P. assisted with the ANN. M.Z. and J.D. assisted with the EBE treatment. H.H., H.H, and Z.C. assisted with the Raman and PL measurements. C.W. assisted with the device fabrication. X.Z. assisted with the electrical property measurements. Y.C., J.W., T.S., L.L., and P.Z. reviewed and edited the manuscript. J. W. supervised all the experiments, calculations, and data collection. All authors contributed to the data interpretation, presentation, and writing of the manuscript.

[Correction added on 18 October 2024 after online publication: Author name, Peng Gao was corrected in author byline and author contributions section.]

## Supporting information



Supporting Information

## Data Availability

The data that support the findings of this study are available from the corresponding author upon reasonable request.
