# Supplementary material for: Two‐Dimensional MoS2‐Based Anisotropic Synaptic Transistor for Neuromorphic Computing by Localized Electron Beam Irradiation
Source: Adv Sci (Weinh). 2024 Oct 16;11(45):2408210. doi: 10.1002/advs.202408210 (PMC11615781; doi:10.1002/advs.202408210)
Supplement: Supplementary file 1 — Supporting Information [file ADVS-11-2408210-s001.docx]

Supporting Information

Two-Dimensional MoS_2_-Based Anisotropic Synaptic Transistor for Neuromorphic Computing by Localized Electron Beam Irradiation

Lei Liu, Gao Peng, Mengru Zhang, Jiadu Dou, Chunsen Liu, Tuo Shi, Hao Huang, Chunlan Wang, Han He, Zijun Chen, Yang Chai, Jianlu Wang, Xuming Zou, Lei Liao*, Jingli Wang* and Peng Zhou*

**
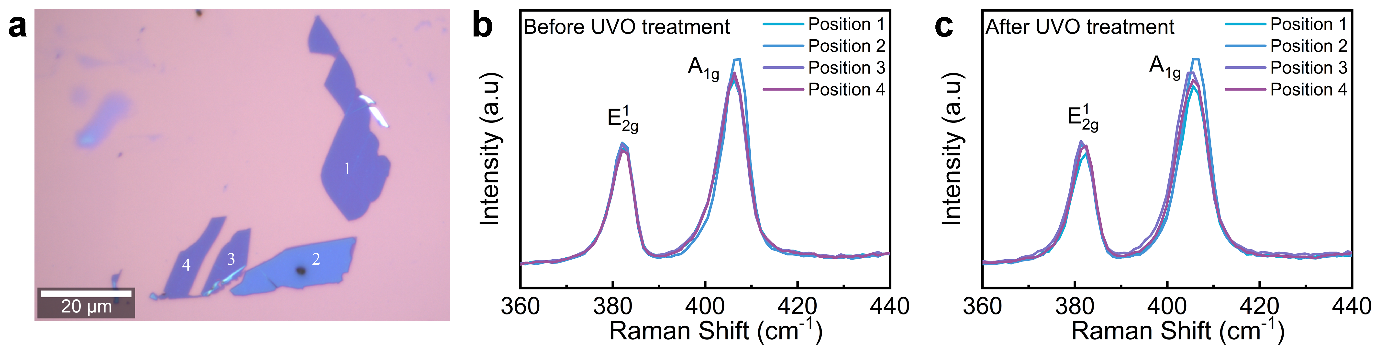
**

**Figure S1.** The Raman spectrum of MoS_2_ thin films before and after UVO treatment. a) Optical image of the MoS_2_ nanoflakes for Raman measurement. b) The Raman spectrum before UVO treatment. c) The Raman spectrum after 90 s UVO treatment.

**Table S1.** The variations of Raman spectrum before and after 90 s UVO treatment.

|  | **FWHM**  **before UVO treatment (cm^-1^)** | | **FWHM**  **after UVO treatment (cm^-1^)** | | **Position**  **before UVO treatment (cm^-1^)** | | **Position**  **after UVO treatment (cm^-1^)** | |
| --- | --- | --- | --- | --- | --- | --- | --- | --- |
| **Position 1** | E^1^_2g_ | 6.09777 | E^1^_2g_ | 6.70115 | E^1^_2g_ | 382.339 | E^1^_2g_ | 381.933 |
|  | A_1g_ | 6.85509 | A_1g_ | 7.45002 | A_1g_ | 406.190 | A_1g_ | 405.813 |
| **Position 2** | E^1^_2g_ | 5.99270 | E^1^_2g_ | 6.12027 | E^1^_2g_ | 382.185 | E^1^_2g_ | 381.820 |
|  | A_1g_ | 6.52153 | A_1g_ | 7.43351 | A_1g_ | 406.805 | A_1g_ | 406.159 |
| **Position 3** | E^1^_2g_ | 6.23007 | E^1^_2g_ | 6.26550 | E^1^_2g_ | 382.223 | E^1^_2g_ | 381.593 |
|  | A_1g_ | 7.18917 | A_1g_ | 8.26336 | A_1g_ | 406.057 | A_1g_ | 405.217 |
| **Position 4** | E^1^_2g_ | 6.22164 | E^1^_2g_ | 6.37718 | E^1^_2g_ | 382.358 | E^1^_2g_ | 381.965 |
|  | A_1g_ | 7.10007 | A_1g_ | 8.38222 | A_1g_ | 406.267 | A_1g_ | 405.793 |

Figure S1a presents the optical image of the MoS_2_ nanoflakes for Raman measurement. Figure S1b and 1c present the Raman spectrum of MoS_2_ nanoflakes before and after UVO treatment, respectively. The half of the maximum (FWHM) and peak positions of E^1^_2g_ and A_1g_ mode are summarized in Table S1. It is observed that all the full-width at the four positions will increase and positions of peaks will blue shift after being treated by UVO, indicating the existence of trap sites.

**
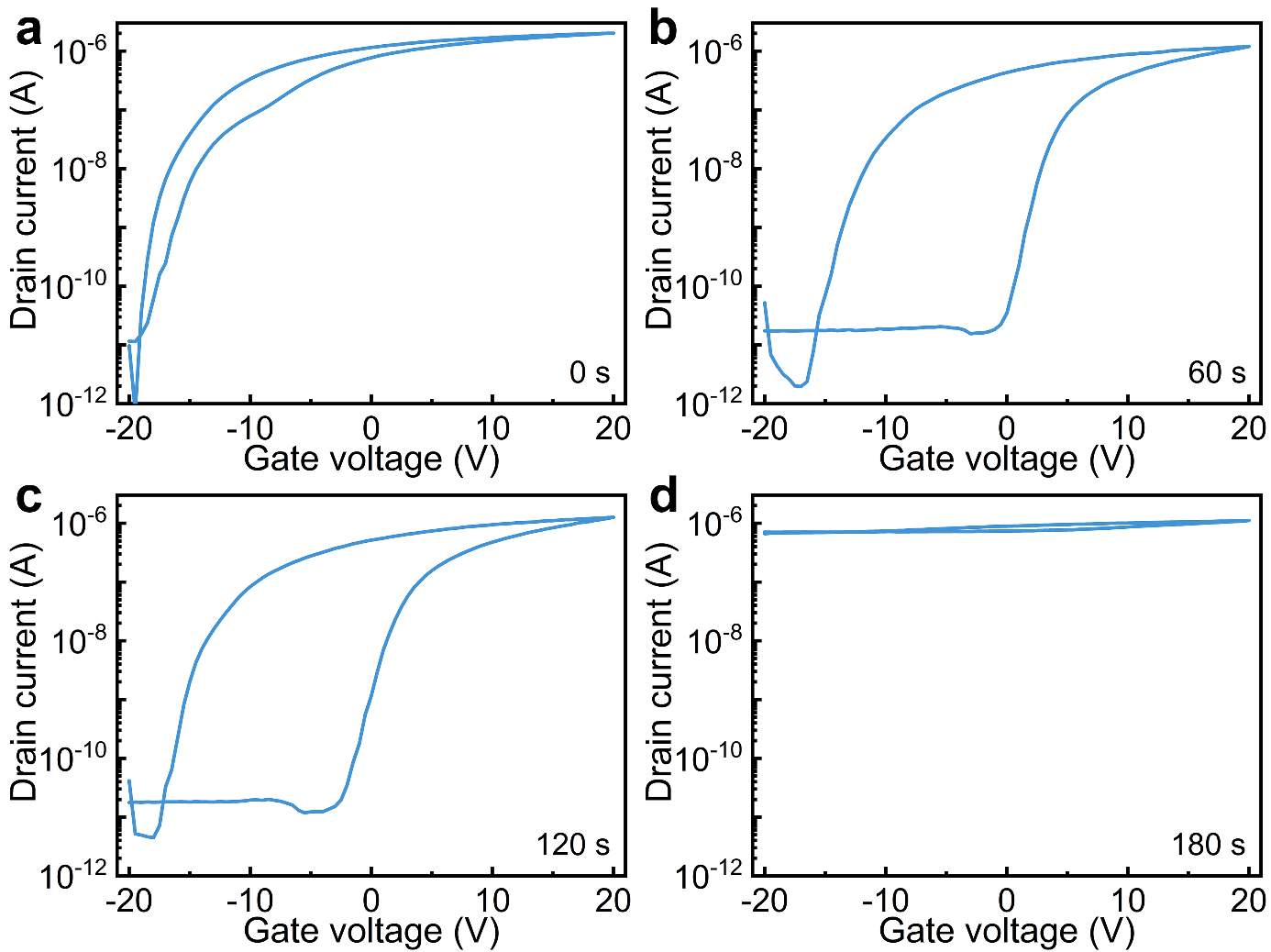
**

**Figure S2.** Transfer characteristics of MoS_2_-based transistor with different irradiation time of UVO treatment at *V_ds_*=0.5 V. a) 0 s. b) 60 s. c) 120 s. d) 180 s.

Figure S2 shows double sweep transfer characteristics of the device with different irradiation time of UVO treatment. Initially, the transfer curve reveals a narrow memory window of 1 V before UVO treatment (Figure S2a). Following a 60 s UVO treatment, the memory window increases to 15 V, suggesting that trap sites are effectively introduced through the use of UVO (Figure S2b). Following a 120 s UVO treatment, the device exhibits a memory window of 14.5 V similar to that of 60 s UVO treatment (Figure S2c). However, after a 180 s UVO treatment, the device loses its current regulation capability due to the excessive trap sites (Figure S2d). Herein, the devices are treated by UVO for 90 s to explore their electrical and optoelectronic properties.

**
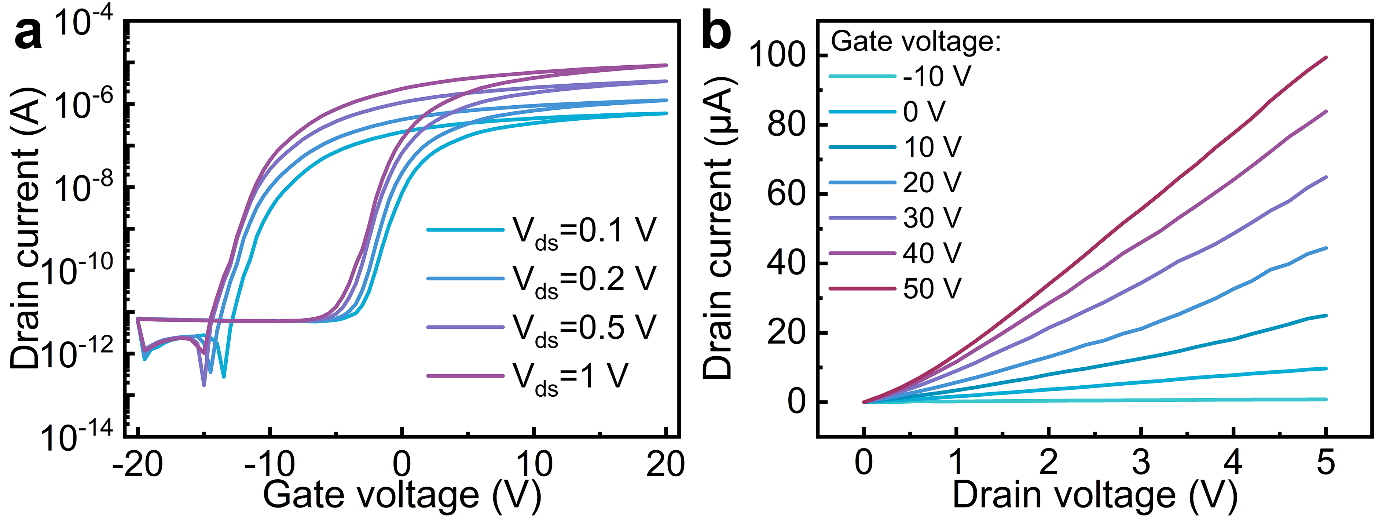
**

**Figure S3.** Basic electrical properties of MoS_2_-based transistor after UVO treatment. a) Transfer curves of the MoS_2_ transistor under different *V_ds_* from 0.1 V to 1 V. b) Output characteristics of the transistor under different *V_gs_* from -10 V to 50 V.

Figure S3a presents the transfer characteristics of MoS_2_-based transistor under various *V_ds_* following UVO treatment. A notable trend is observed as the *V_ds_* increases from 0.1 V to 1 V, transfer curve progressively shifts towards the negative direction, concurrently with an increase in drain current. Figure S3b shows corresponding output characteristics under varying *V_gs_*. As the *V_gs_* rises from -10 V to 50 V, the drain current will also become higher.

**
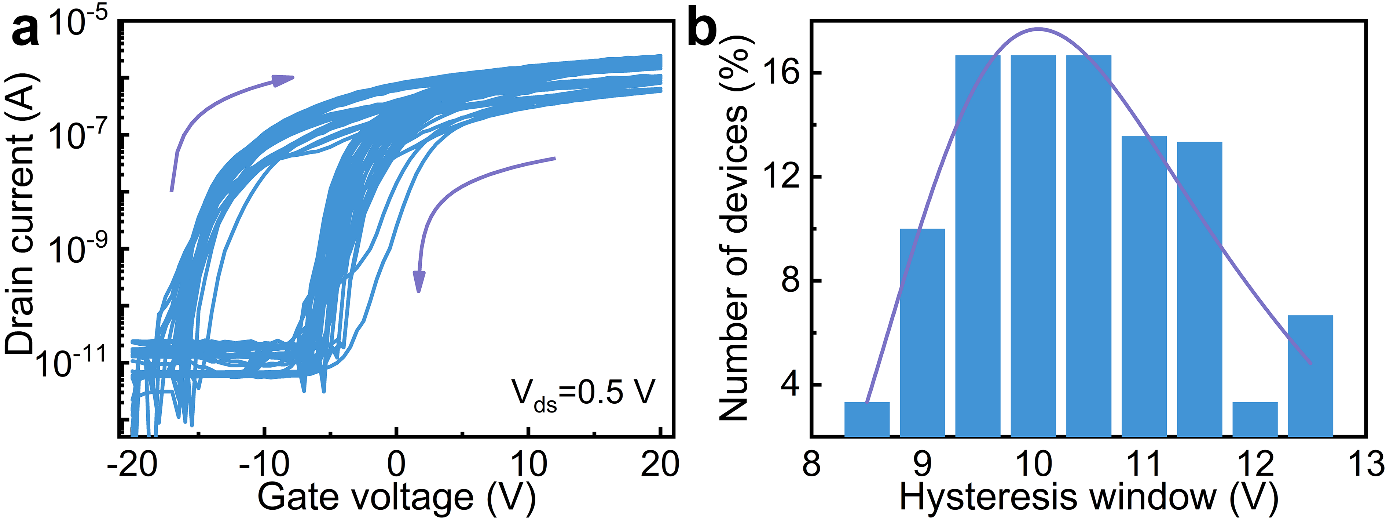
**

**Figure S4.** The statistic characteristics of 30 MoS_2_ transistors after UVO treatment. a) The transfer curves at drain voltage *V_ds_*=0.5 V of these devices. b) Histogram of the hysteresis window for these devices and the Gaussian fit (solid lines) based on the statistical data.

Figure S4a shows the 30 typical transfer curves with the *V_ds_* of 0.5 V after UVO treatment. During the *V_gs_* sweeping between -20 V and 20 V, these devices exhibit expanded memory windows. Figure S4b presents the statistic hysteresis window with an average value of about 10 V, indicating the stable enhanced electrical hysteresis performance after UVO treatment.

**
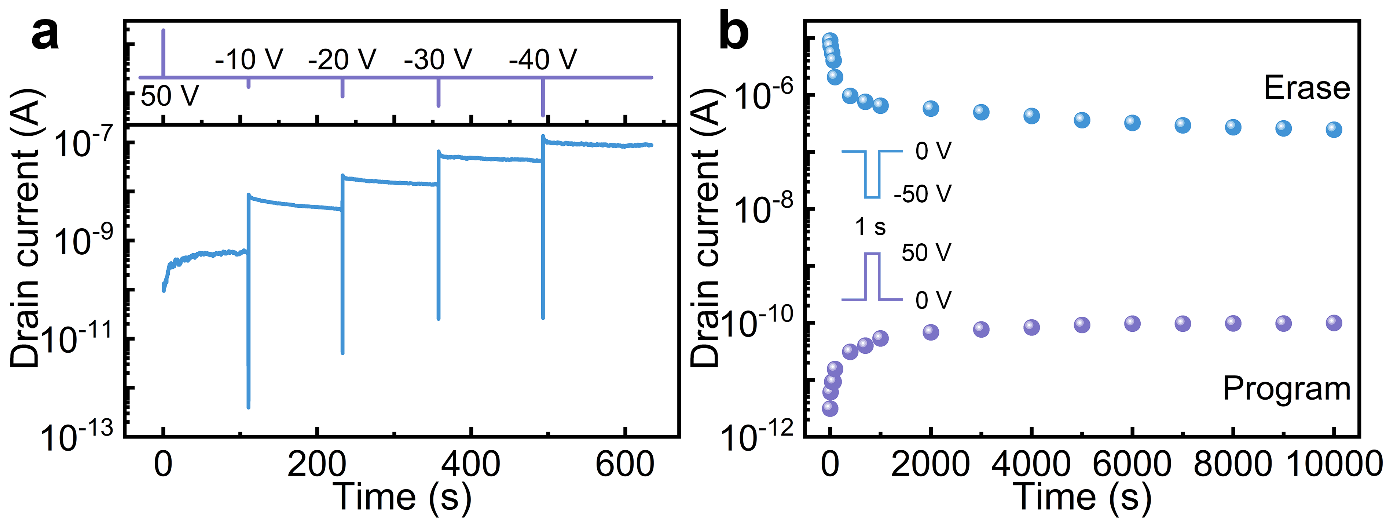
**

**Figure S5.** The memory properties of MoS_2_-based transistor. a) Continuous change of drain current modulated by different gate voltage pulse. b) Retention property of MoS_2_ transistor.

Continuous changes of drain current under various *V_gs_* pulses are demonstrated in Figure S5a. Initially, the drain current is modulated to a lower state under the *V_gs_* pulse of +50 V (pulse width: 0.2 s). Subsequently the drain current increase to various higher state under the *V_gs_* pulses of -10 V, -20 V, -30 V and -40 V (pulse width: 1 s) in sequence, illustrating the multilevel storing property in the MoS_2_-based transistor. Subsequently, the device is modulated to high resistance state (HRS)/low resistance state (LRS) by applying the pulse of +50 V/-50 V, respectively. Figure S5b displays the retention characteristics of HRS and LRS with a high on/off ratio of over 2×10^3^ after 10000 s, indicating the exceptional nonvolatile feature of the device.

**
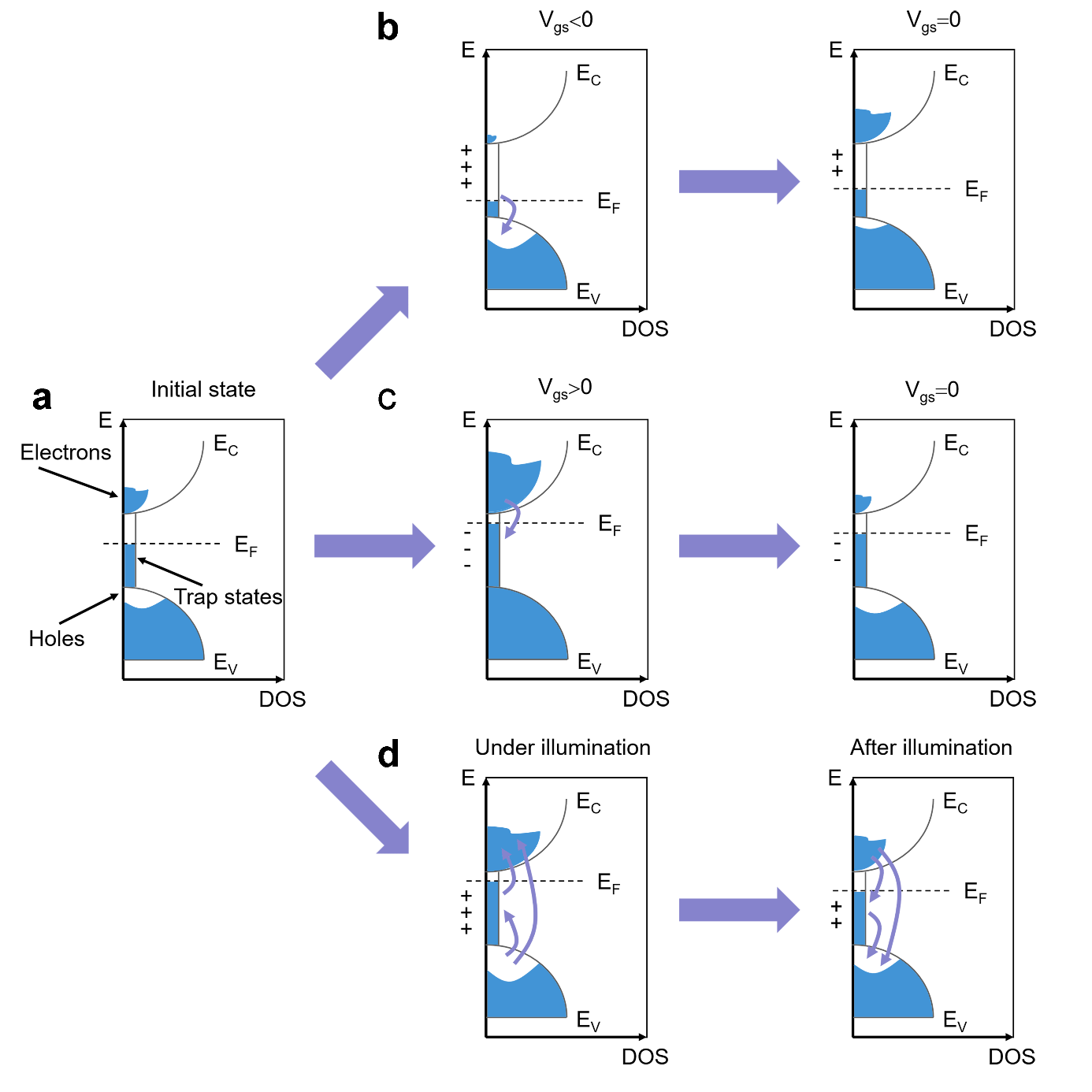
**

**Figure S6.** The memory mechanism of MoS_2_-based transistor treated by UVO. Schematic of the band structure of the MoS_2_ transistor a) at initial state, b) under negative *V_gs_*, c) under positive *V_gs_* and d) under illumination.

To explain the memory mechanism of MoS_2_-based transistor treated by UVO, the band diagrams are illustrated in Figure S6. Comparing to pristine MoS_2_, there are many UVO-induced localized trap sites in the bandgap of MoS_2_ after UVO treatment. These trapping sites, which serve as ambipolar states to capture charges, are dispersed over a wide energy range in the bandgap. Initially, it is considered that all donor-type traps are populated by electrons and all acceptor-type traps are empty, as presented in Figure S6a. When under the negative *V_gs_*, the Fermi level (E_F_) will be closer to the valence band (E_V_) and the electrons are released by the trapping sites over E_F_. As a result, these traps will be positively charged, inducing additional electrons in the E_C_ and raising channel current. After setting the *V_gs_* to 0 V, these traps of losing electrons will remain for a long period, so channel current is also held at a high state (Figure S6b). When under the positive *V_gs_*, the E_F_ is closer to the conduction band (E_C_) and the electrons are captured by the trapping sites below E_F_, inducing the less electrons in E_C_. After the *V_gs_* is adjusted to 0 V, these traps of capturing electrons will also continue to exist for a long time, resulting reduced channel current (Figure S6c). When illuminated, photoinduced carriers would be generated in MoS_2_ layer. One part of the photogenerated electrons will be captured by traps and the other part will enter the E_C_. These photogenerated electrons will also persist for a period of time after illumination, which will induce increasing current in channel (Figure S6d).

**
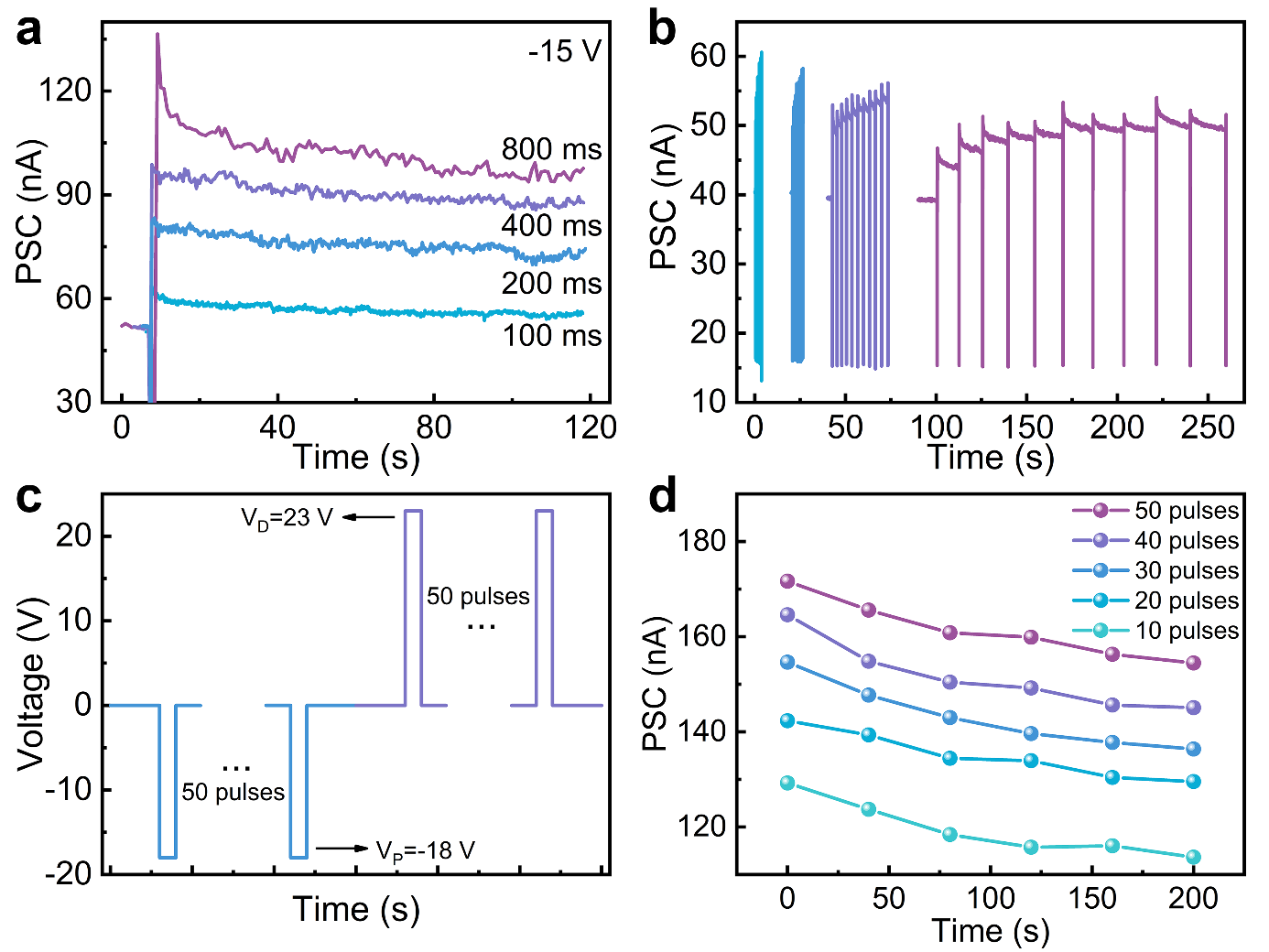
**

**Figure S7.** The synaptic behaviors of MoS_2_-based transistor. a) PSC under negative pulses with different pulse widths of 100, 200, 400, and 800 ms. b) Spike frequency-dependent responses of PSC. c) Schematic diagram of pulse sequences to induce LTP and LTD. d) Retention characteristics of the multilevel resistance states after different number of pulses.

Figure S7a presents the EPSC behaviors by applying pulses with different widths and Figure S7b shows the EPSC behaviors by applying pulses with different frequency. These results demonstrate that the bigger synaptic weight change is both induced by the larger width and frequency of pulse. Figure S7c presents the pulse sequences employed to trigger LTP and LTD. For LTP, 50 negative pulses (-18 V) with a pulse width of 60 ms and an interval of 200 ms are applied to enhance PSC. Subsequently, 50 positive pulses (23 V) are used as depression stimulation. The pulse width and interval are 30 and 600 ms, respectively. The retention property after pulse sequence is also investigated. The retention times are shown in Figure S7d by applying different number of pulses to the artificial synapse with initial PSC of 40 nA. The PSC will remain at a higher current state over 100 s after applying theses pulses, confirming that long-term plasticity is realized under these pulses.


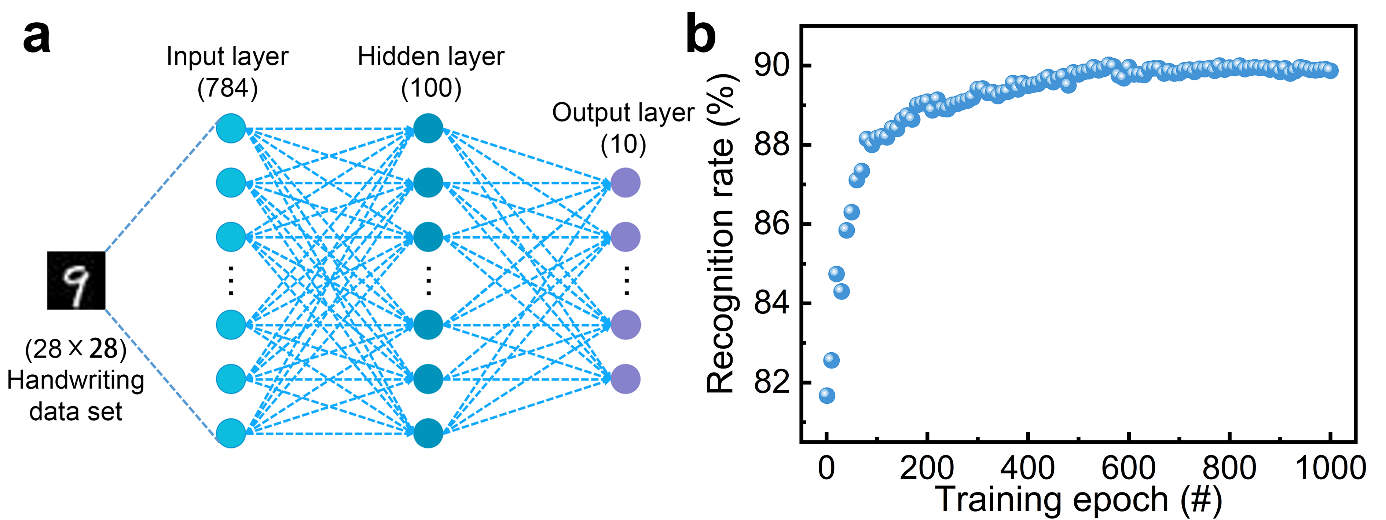


**Figure S8.** The pattern recognition by ANN based on MoS_2_ synaptic transistor. a) The schematic of ANN based on MoS_2_ synaptic transistor. b) The recognition accuracy as a function of training epoch (0–1000).

The three-layer artificial neural network (ANN) is shown schematically in Figure S8a, including input layer with 784 input neurons, hidden layer with 100 hidden neurons and output layer with 10 output neurons. The connection strength between neighboring neurons is modulated according to the LTP and LTD updating process of the MoS_2_ artificial synapse. As demonstrated in Figure S8b, the recognition accuracy of 89.87% is eventually realized through 1000 training epochs.


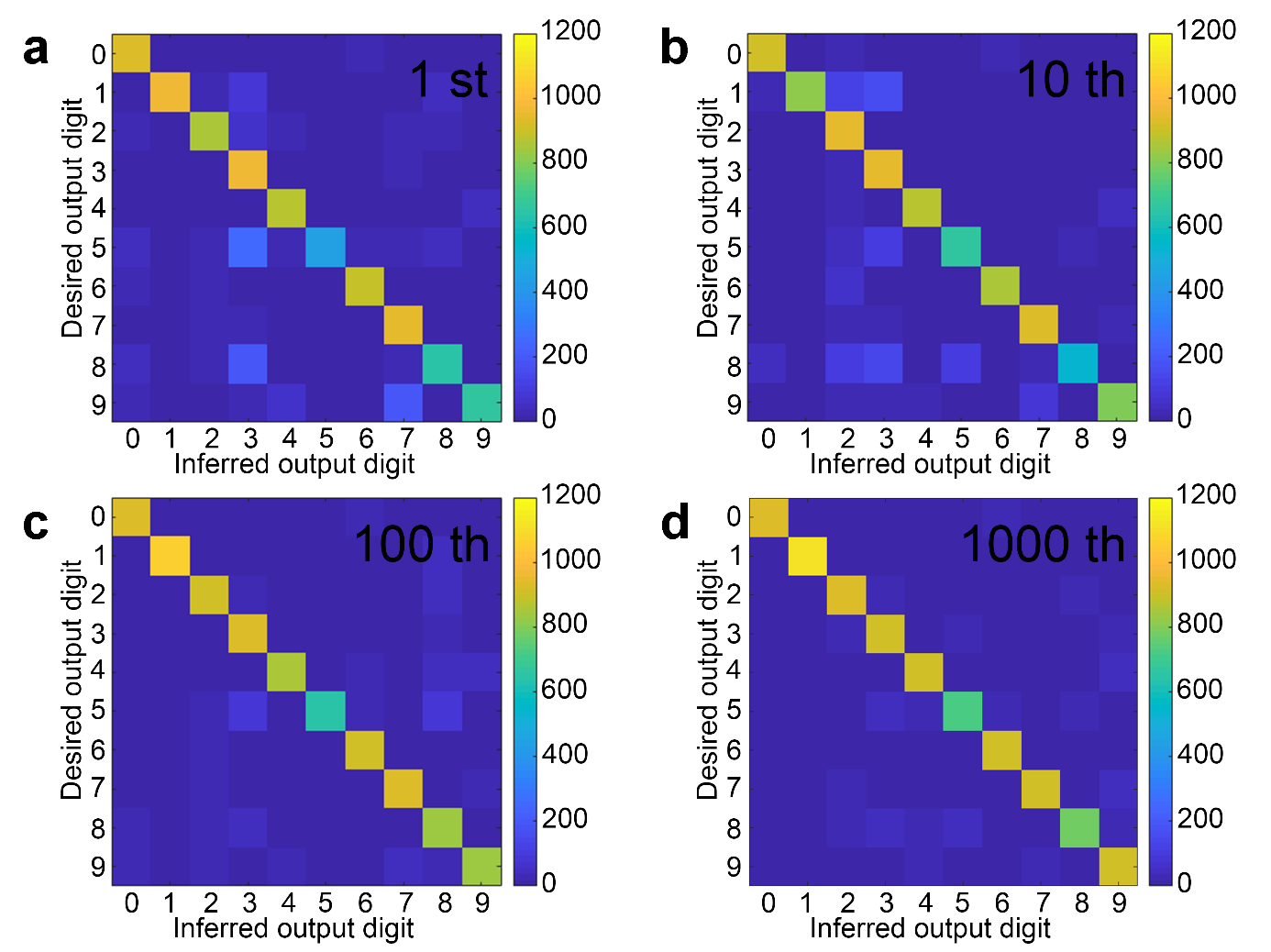


**Figure S9.** Confusion matrices of training results a) under the 1st epoch, b) under the 10th epoch, c) under the 100th epoch and d) under the 1000th epoch.

Figure S9 displays the training results of pattern recognition after 1, 10, 100, and 1000 training epochs, demonstrating improved recognition capability during training process.


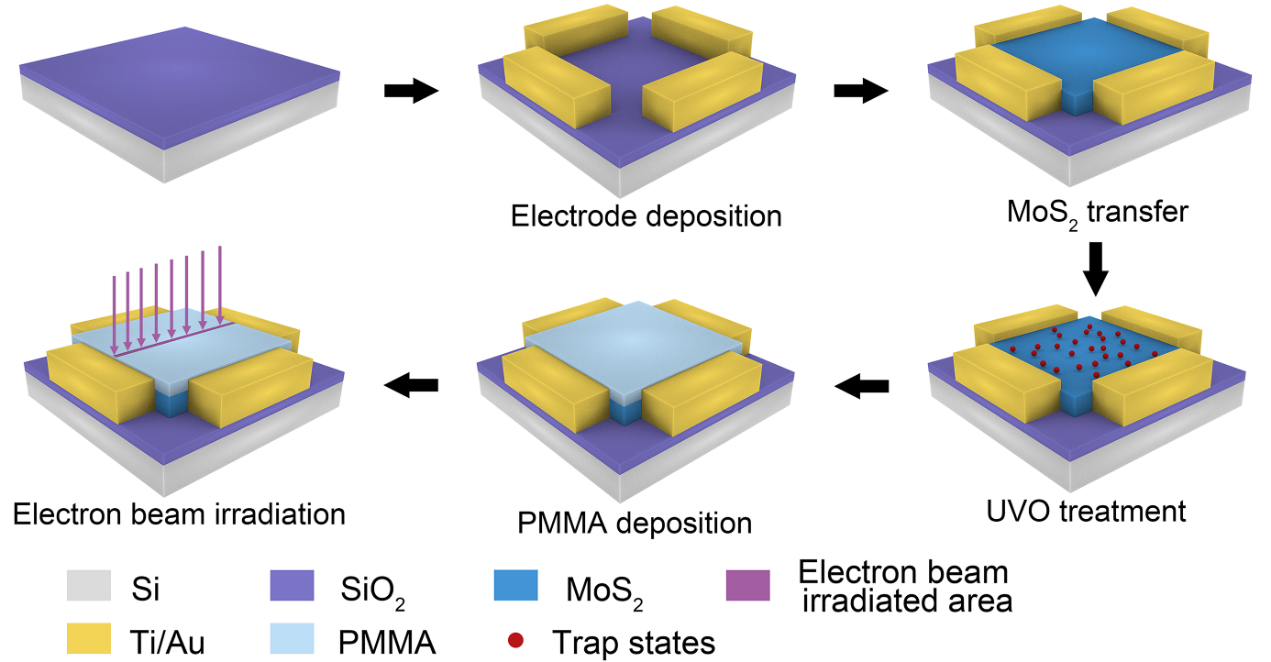


**Figure S10.** Schematic of the device fabrication procedures.

Figure S10 shows schematic of the device fabrication procedures. Firstly, the source and drain electrodes (Ti/Au, 7 nm/30 nm) are fabricated on the Si wafer with 100 nm SiO_2_ via photolithography, electron-beam evaporation and lift-off process. Following this, MoS_2_ nanoflakes are mechanically exfoliated onto the top of source and drain electrodes as channel. Subsequently, device is annealed at 120 ℃ in a vacuum environment for 2 hours. Then device is treated by UVO at 100 ℃ for 90 s to generate trap states. Next, PMMA film is prepared on the MoS_2_-based transistor through the spin-coating process of 600 rpm for 10 s and 6000 rpm for 60 s. PMMA film is heated at 180 ℃ for 3 min to dry solution. Finally, device is treated by EBI with a predefined pattern. The area dose of the EBI treatment is 150 μC/cm^2^.


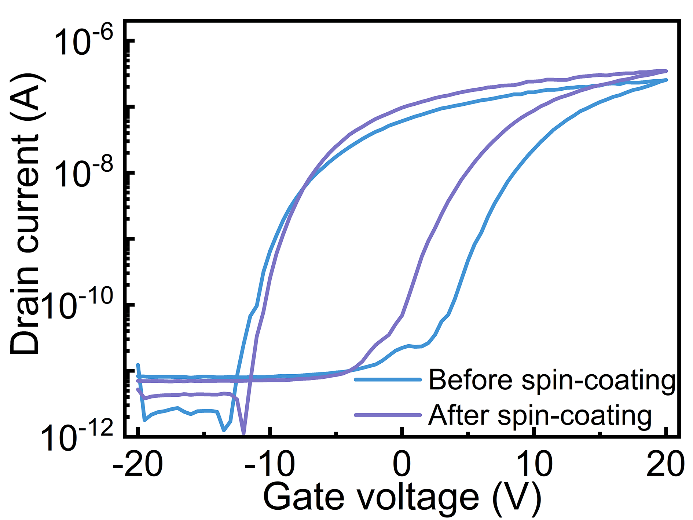


**Figure S11.** Comparison of transfer curves of the device before and after the spin-coating process with PMMA.

Figure S11 presents transfer curves of the device before and after the spin-coating process with PMMA. It is observed that drain current increases and the hysteresis window decreases following the spin-coating process with PMMA. These changes are primarily attributed to the baking process that follows PMMA coating. Specifically, the drying process at 180 °C improves the contact between Au electrodes and MoS_2_ channel, and reduces defects at the interface between SiO_2_ layer and MoS_2_ layer. As a result, we see an increase in drain current and a reduction in hysteresis window.


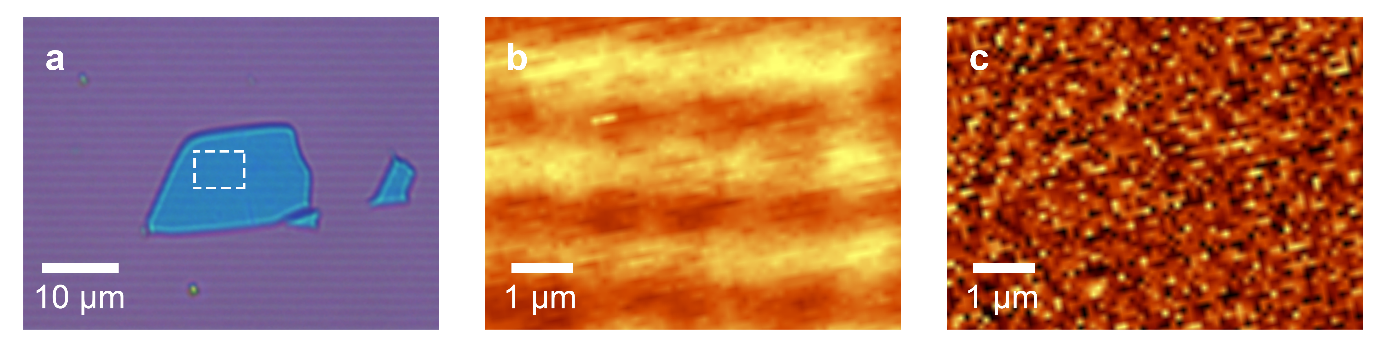


**Figure S12.** PL and Raman results of MoS_2_ nanoflake with PMMA film after EBI treatment with predefined pattern. a) Optical image of the MoS_2_ nanoflake with PMMA film after EBI treatment. b) Mapping image of PL intensity of the MoS_2_ nanoflake. c) Mapping image of Raman FWHM of the MoS_2_ nanoflake.

As presented in Figure S12a, The MoS_2_ nanoflake is treated by the EBI with predefined pattern to investigate the effect of EBI. Subsequently, the PL and Raman properties are measured. As in the case of the PL peak center, variations in PL intensity are also observed between the irradiated area and unirradiated area, as shown in Figure S12b. In addition, the mapping image of Raman FWHM (Figure S12c) shows that the Raman FWHM remains virtually unchanged before and after EBI treatment. This indicates that compared to UVO treatment, the effect of EBI is less dramatic.


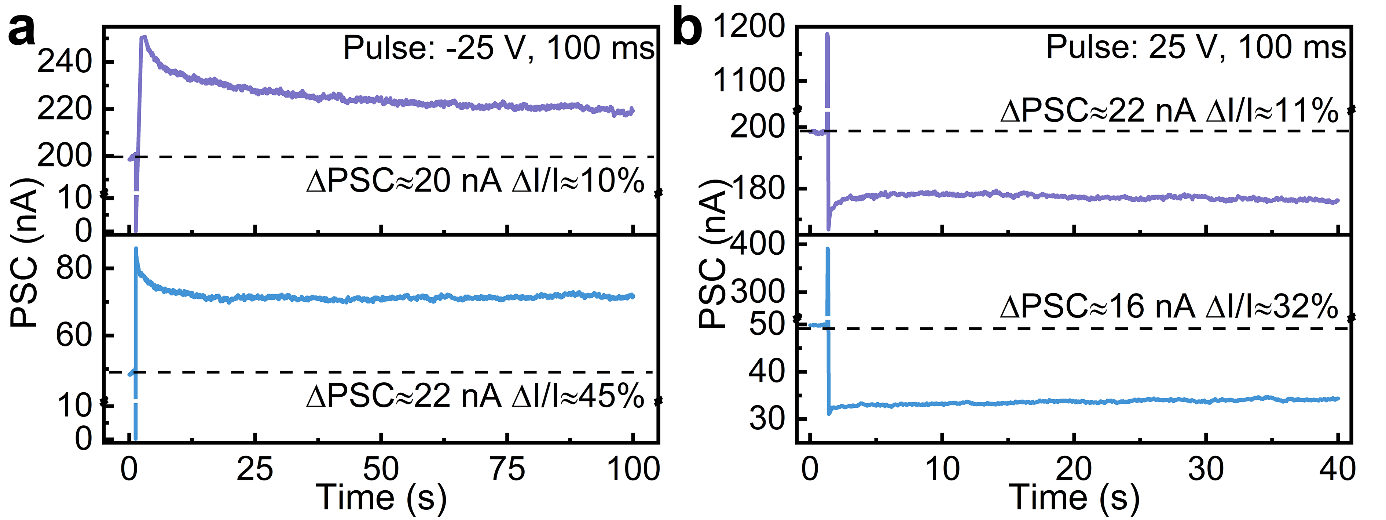


**Figure S13.** The anisotropic synaptic behaviors of the MoS_2_-based multiterminal transistor. Anisotropic PSC change for synapse 1 and synapse 2 under a) a negative voltage pulse and b) a positive voltage pulse.

Figure S13a presents the EPSC response of synapse 1 and synapse 2 to the negative presynaptic signal (-25 V, 50 ms). Although ΔPSC is similar in synapse 1 (22 nA) and synapse 2 (20 nA), it is observed that the change of PSC is 4.5 times larger in synapse 1(45%) than that in synapse 2 (10%). The IPSC response of synapse 1 and synapse 2 to the positive presynaptic signal (25 V, 50 ms) is also shown in Figure S13b. Similar to the EPSC response, ΔPSC is 16 nA in synapse 1 and 22 nA in synapse 2, and the change of PSC is 2.9 times larger in synapse 1 (32%) than that in synapse 2 (11%).


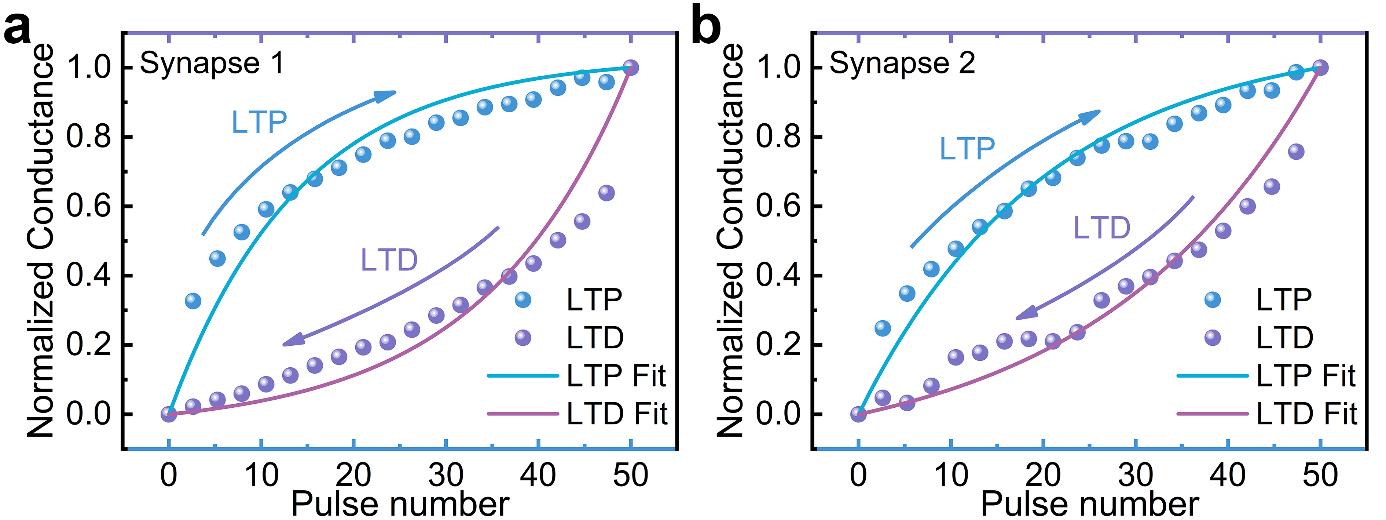


**Figure S14.** The fitted nonlinearity coefficient of LTP and LTD for a) synapse 1 and b) synapse 2.

Figure S14 shows fitted nonlinearity coefficient of synapse 1 and synapse 2. After localized EBI, the device displays anisotropic NLF, with different coefficients along two directions. In direction 1, the NLF is 3.89 for LTP and 3.61 for LTD. While in direction 2, it is 3.04 for LTP and 2.72 for LTD. Compared to NLF before EBI (3.19 for LTP and 4.34 for LTD), synapse 1 shows less change, whereas synapse 2 demonstrates an improvement in NLF.


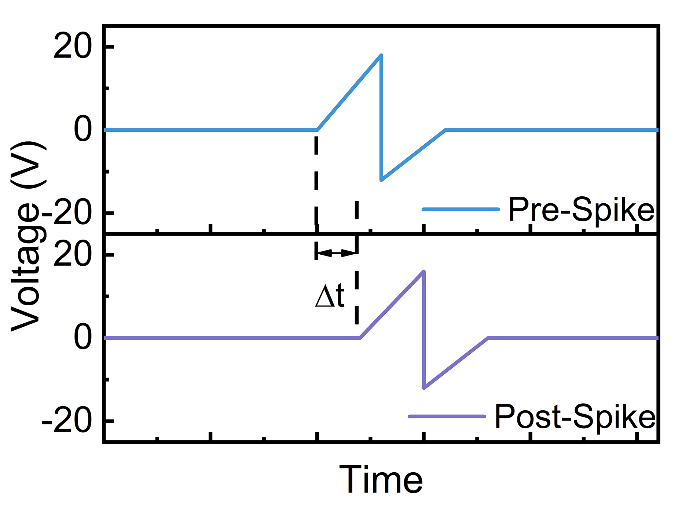


**Figure S15.** Pre- and post-spikes used in STDP.

As shown in Figure S15, pre- (*V_pre-spike_*) and post-synaptic spikes (*V_post-spike_*) are designed and their net efficient spike (*V_net_*=*V_pre-spike_*-*V_post-spike_*) is applied at gate to realize STDP characteristic. The PSC is read 1 s before and 5 min after the spiking pair to obtain the change of PSC.


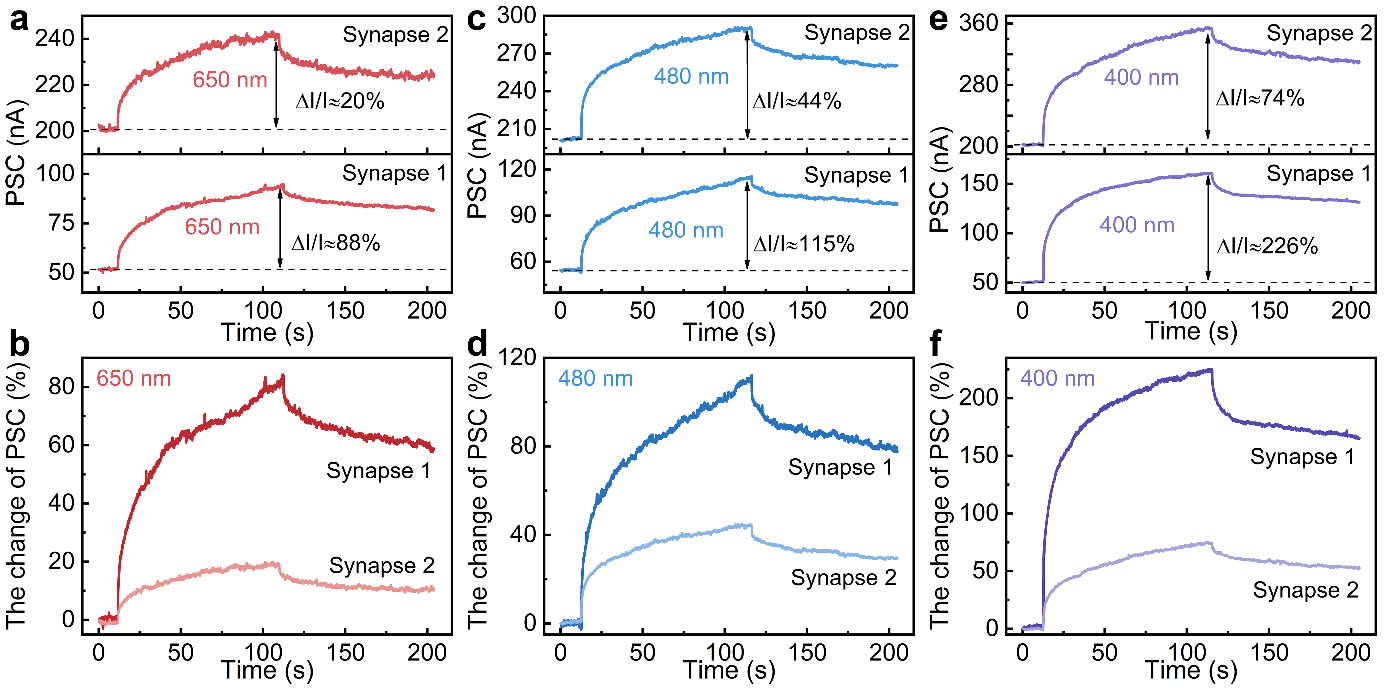


**Figure S16.** The different responses of PSC for synapse 1 and synapse 2 under illumination with different wavelengths. a) The PSC of synapse 1 and synapse 2 under illumination of 650 nm. b) The change of PSC of synapse 1 and synapse 2 under illumination of 650 nm. c) The PSC of synapse 1 and synapse 2 under illumination of 480 nm. d) The change of PSC of synapse 1 and synapse 2 under illumination of 480 nm. e) The PSC of synapse 1 and synapse 2 under illumination of 400 nm. f) The change of PSC of synapse 1 and synapse 2 under illumination of 400 nm.

The optical responses for the two synapses under illumination with various optical wavelengths are presented in Figure S16. Equally, drain current is read through *V_ds_* of 0.1 V. The PSC and change of PSC of synapse 1 and synapse 2 are shown under illumination with different wavelengths of 650 nm (Figure S16a and Figure S16b), 480 nm (Figure S16c and Figure S16d) and 400 nm (Figure S16e and Figure S16f). It is observed the change of response current to light stimulation along direction 1 is larger than that along direction 2.


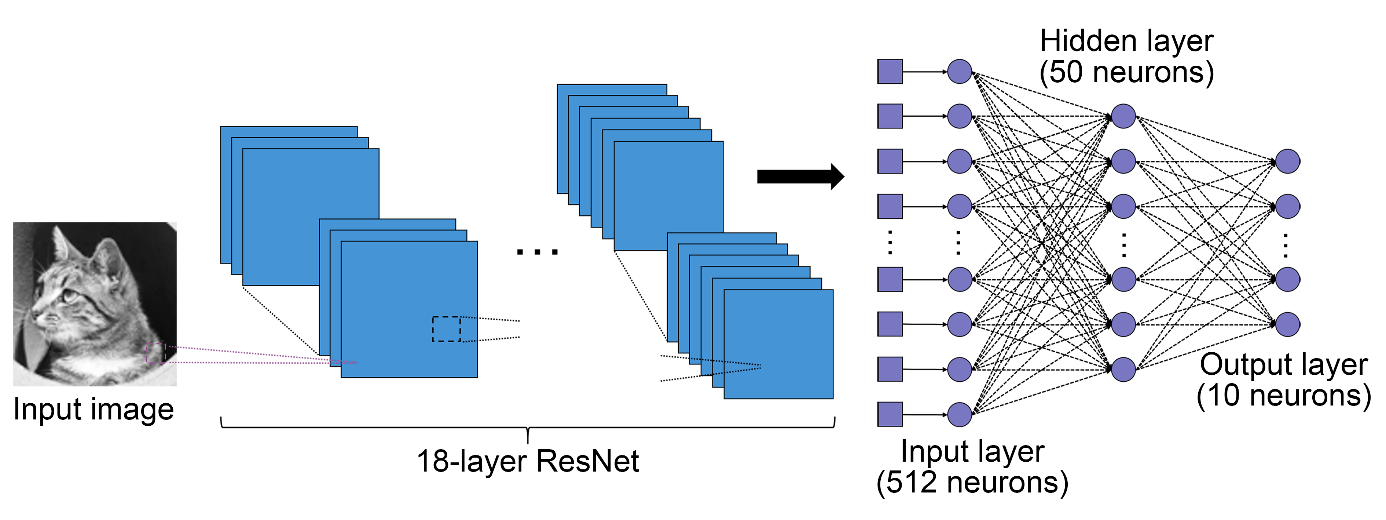


**Figure S17.** Schematic diagram of ANN for image recognition.

Figure S17 demonstrates the schematic of ANN used for this task. Feature maps are extracted from input images with 96×96 pixels through an 18-layer convolutional neural network (ResNet18) and then passed to the fully connected network for classification. Fully connected network comprises 512 input neurons, 50 hidden neurons and 10 output neurons, with synaptic weights updated according to the LTP/LTD properties of MoS_2_-based transistors in different directions. The 10 output neurons represent various object categories, and recognition accuracy is assessed by matching output categories to image contents. The dataset includes 500 images per category, with 90% used for training and 10% for testing.

During the first 56 epochs of training process, recognition rates are improved from 19.4% to 86.4% for synapse 1 and from 18.2% to 86.0% for synapse 2. In this process, the recognition accuracy for synapse 1 is superior to that for synapse 2. From epochs 57 to 200, recognition rates are increased to 88% (synapse 1) and 93.8% (synapse 2), respectively. In this process, the recognition accuracy for synapse 2 is higher to that for synapse 1. These results illustrate the ANN based on synapse 1 enables faster learning rate, while the ANN based on synapse 2 ultimately achieves higher accuracy in the long run.


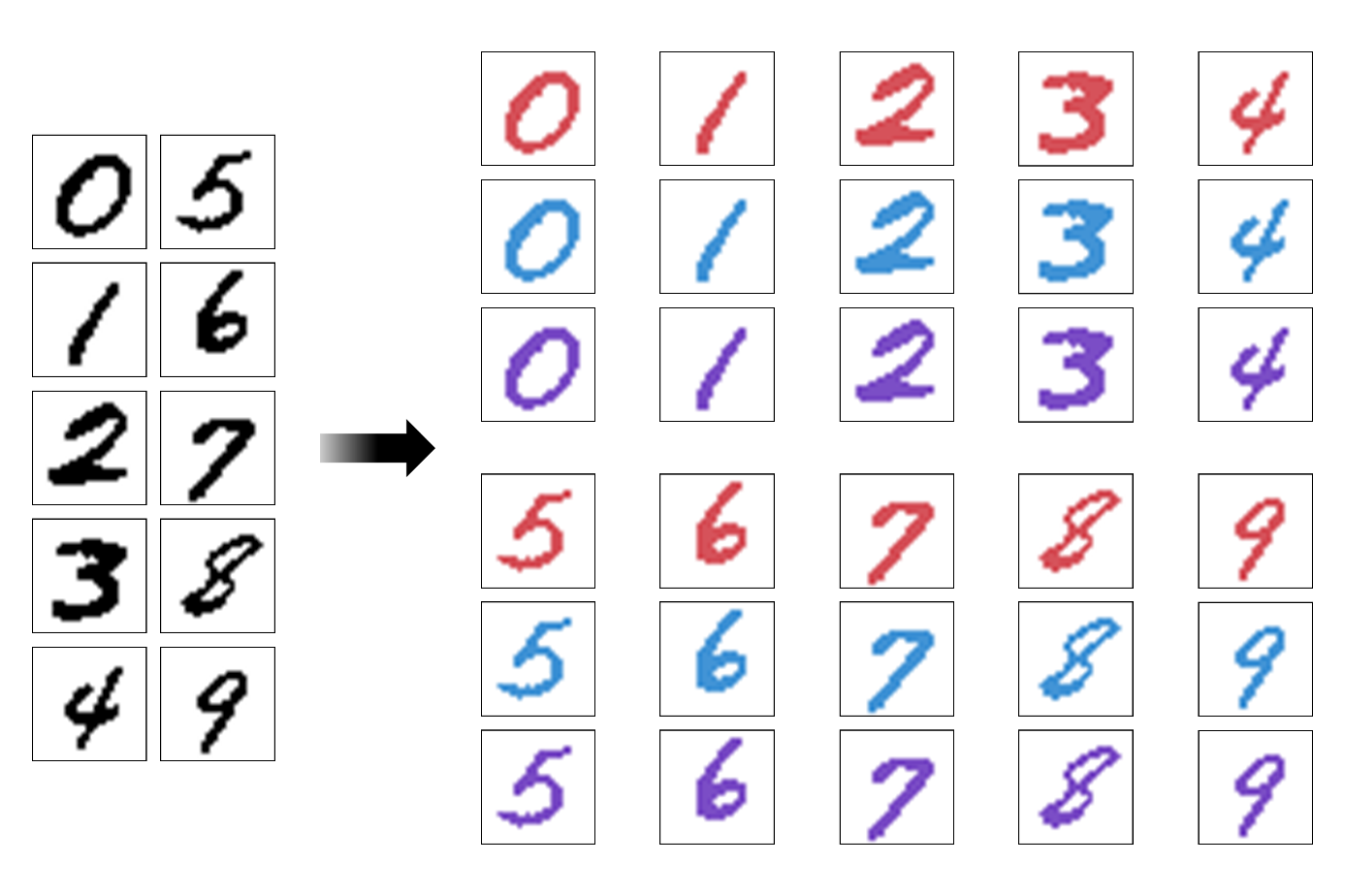
**Figure S18.** Handwritten datasets of original images and colored-digit images without distracting background for colored-digit recognition.


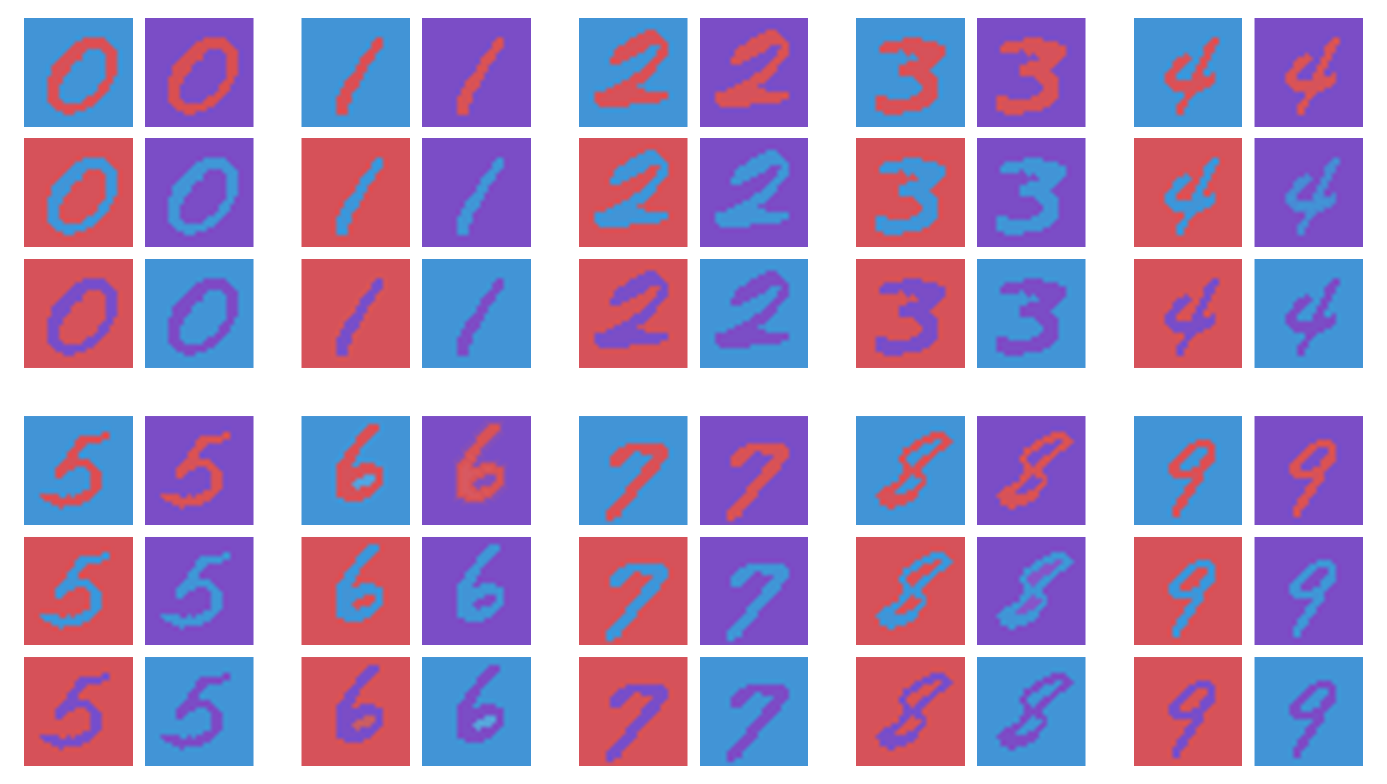


**Figure S19.** Handwritten datasets of colored-digit images with distracting background for colored-digit recognition.

Figure S18 and Figure S19 illustrate the handwritten datasets of colored-digit images without and with distracting background. To create these datasets, images from the original MNIST dataset are randomly selected, and the digit and background regions are distinguished based on pixel intensity values. The digit regions are colored using one of three randomly chosen colors (red, blue, or purple) to generate the colored-digit MNIST dataset. For images with distracting backgrounds, the digit and background regions are each assigned different colors from the chosen colors.

For each colored digit, approximately 2000 images are prepared as training dataset and about 400 images are prepared for the pattern recognition test. After simulation, the 30 output neurons represent red, blue, and purple 0-9 (R0-R9, B0-B9 and P0-P9) to export recognition results for colored digits.

**Note S1.** **Estimation of Nonlinearity.**

The behavior of nonlinear weight update is evaluated by the following equation:

$$\begin{aligned} G_{LTP}=B\left( 1-e^{\left( -\frac{P}{A} \right)} \right)+G_{min}\text{ }\text{}\text{S}\text{}\text{} \end{aligned}$$

$$\begin{aligned} G_{LTD}=-B\left( 1-e^{\left( \frac{P-P_{max}}{A} \right)} \right)+G_{max} \text{(S2)} \end{aligned}$$

$$\begin{aligned} B=\frac{G_{max}-G_{min}}{1-e^{-\frac{P_{max}}{A}}} \text{(S3)} \end{aligned}$$

$$\begin{aligned} \alpha=\frac{1.726}{A+0.162} \text{(S4)} \end{aligned}$$

where *G_max_*, *G_min_* and *P_max_* are the maximum conductance, minimum conductance, and the maximum pulse number to adjust device to the minimum and maximum conductance states in LTP and LTD. A is the parameter to determine the nonlinear behavior of the LTP and LTD and α is the nonlinearity factor to evaluate the behavior of nonlinear weight update.

**Note S2. The relationship between *W_Change_* and *Δt* in asymmetric Hebbian STDP.**

In asymmetric Hebbian STDP, LTP will arise when the presynaptic spike precedes the postsynaptic one in arriving (*Δt*>0). Otherwise, LTD will arise in the opposite operation (*Δt*<0). A bigger change of PSC will be generated by the shorter time intervals.

Additionally, the change of synaptic weight (*W_Change_*) can be fitted by the following relation with *Δt*:

$$\begin{aligned} W_{Change}=\left\{ \begin{aligned} A_{+}\exp\left( -\Delta t/\tau_{+} \right), \Delta t>0 \\ {-A}_{-}\exp\left( \Delta t/\tau_{-} \right), \Delta t<0 \end{aligned} \right. \text{(S5)} \end{aligned}$$

where A_+_ and A_−_ are scaling factors, τ_+_ and τ_−_ are time constants.
